# A Combined Phytochemistry and Network Pharmacology Approach to Reveal the Effective Substances and Mechanisms of Wei-Fu-Chun Tablet in the Treatment of Precancerous Lesions of Gastric Cancer

**DOI:** 10.3389/fphar.2020.558471

**Published:** 2020-11-18

**Authors:** Huijun Wang, Ruoming Wu, Dong Xie, Liqin Ding, Xing Lv, Yanqin Bian, Xi Chen, Bahaji Azami Nisma Lena, Shunchun Wang, Kun Li, Wei Chen, Guan Ye, Mingyu Sun

**Affiliations:** ^1^ Key Laboratory of Liver and Kidney Diseases (Ministry of Education), Institute of Liver Diseases, Shuguang Hospital, Shanghai University of Traditional Chinese Medicine, Shanghai, China; ^2^ Central Research Institute, Shanghai Pharmaceuticals Holding Co., Ltd., Shanghai, China; ^3^ The MOE Key Laboratory for Standardization of Chinese Medicines and the SATCM Key Laboratory for New Resources and Quality Evaluation of Chinese Medicines, Institute of Chinese Materia Medica, Shanghai University of Traditional Chinese Medicine, Shanghai, China; ^4^ Shanghai Zhonghua Pharmaceutical Co., Ltd., Shanghai, China; ^5^ Huqingyutang Chinese Medicine Modernization Research Institute of Zhejiang Province, Hangzhou, China

**Keywords:** Wei-Fu-Chun tablet, precancerous lesions of gastric cancer, network pharmacology, effective substances and mechanism, UHPLC-ESI-Q-TOF/MS

## Abstract

Wei-Fu-Chun (WFC) tablet is a commercial medicinal product approved by China Food and Drug Administration, which is made of *Panax ginseng* C.A.Mey., *Citrus aurantium* L., and *Isodon amethystoides* (Benth.). WFC has been popularly used for the treatment of precancerous lesions of gastric cancer (PLGC) in clinical practice. In this study, a UHPLC-ESI-Q-TOF/MS method in both positive and negative ion mode was employed to rapidly survey the major constituents of WFC. 178 compounds including diterpenoids, triterpenes, sesquiterpenes, flavonoids, saponins, phenylpropanoids, lignans, coumarins, organic acids, fatty acids, quinones, and sterols, were identified by comparing their retention times, accurate mass within 5 ppm error, and MS fragmentation ions. In addition, 77 absorbed parent molecules and nine metabolites in rat serum were rapidly characterized by UHPLC-ESI-Q-TOF/MS. The network pharmacology method was used to predict the active components, corresponding therapeutic targets, and related pathways of WFC in the treatment of PLGC. Based on the main compounds in WFC and their metabolites in rat plasma and existing databases, 13 active components, 48 therapeutic targets, and 61 pathways were found to treat PLGC. The results of PLGC experiment in rats showed that WFC could improve the weight of PLGC rats and the histopathological changes of gastric mucosa partly by inhibiting Mitogen-activated protein kinase (MAPK) signaling pathway to increase pepsin secretion. This study offers an applicable approach to identify chemical components, absorbed compounds, and metabolic compounds in WFC, and provides a method to explore bioactive ingredients and action mechanisms of WFC.

## Introduction

Traditional Chinese medicine (TCM) is based on the principle of holism, that is, all the body systems are interconnected. Thus, TCM treatments can be multi-target, multi-link, and with minimal side-effects in the prevention and treatment of precancerous lesions of gastric cancer (PLGC), among other diseases. The Wei-Fu-Chun (WFC) tablet, a well-known Chinese herbal preparation, is composed of three herbs: *P. ginseng* (HS: 131 g), *C. aurantium* (ZQ: 250 g), and *I. amethystoides* (XCC: 2,500 g). Dosage of these herbs was derived from *Chinese Pharmacopoeia*, *2020 edition*. In the first part of this edition, WFC was mentioned to tonify spleen qi, promote blood circulation and detoxification, and to have been employed to treat PLGC in clinic for many years (Jin et al., 2015). PLGC is a group of diseases with pathological features of intestinal metaplasia or dysplasia based on chronic atrophic gastritis (Correa et al., 1975). PLGC is an important stage in the development of superficial gastritis to gastric cancer. According to the Chinese Cancer Statistics released by the Chinese Cancer Registry, the mortality rate of gastric cancer ranked second in China, following lung cancer. (Chen et al., 2016). Therefore, clinically halting the progression of PLGC to gastric cancer or reversing it has been the focus of current academic research. Some studies investigated the mechanisms of WFC to treat gastritis *in vitro* or *in vivo* (Zhao et al., 2012; Huang et al., 2014). However, no studies on the action mechanism of WFC in the treatment of PLGC have been conducted so far. With the prominence of network pharmacology in system biology, this distinct and novel approach to the study of complex analytical systems is becoming more widely known and more frequently used in the field of drug research. The role of network pharmacology includes uncovering the functions of TCM, providing scientific evidence for TCM, and establishing TCM as a scientifically-proven field (Ma et al., 2016). Here, we attempted to explore the action mechanisms of WFC in the treatment of PLGC using network pharmacology and experimental studies.

In this paper, a reliable and rapid ultra-high performance liquid chromatography coupled with electrospray ionization quadrupole time-of-flight mass spectrometry (UHPLC-ESI-Q-TOF/MS) method was established to profile complex compounds in WFC and describe their absorption behavior and metabolites in plasma samples after oral administration of WFC. The network pharmacology method was used to construct the pharmacology-network of the “origins-components-targets-pathways” of WFC to systematically predict the active components, targets, and signaling pathways that may treat PLGC. In addition, animal experiment was used to verify the prediction and clarify the mechanisms of action. This study provides a reference for further research and exploration of pharmacodynamics material basis and mechanisms of WFC.

## Experimental Materials and Methods

### Chemicals and Reagents

Drug samples of WFC were obtained from Huqingyutang Pharmaceutical Co., Ltd (Hangzhou, China, batch No. 17066174). HPLC-grade acetonitrile, methanol, and formic acid were purchased from Fisher Scientific (Santa Clara, United States). Ultrapure water was prepared using a Millipore Alpha-Q water system (Millipore, United States). All other reagents were of analytical grade. For animal experiments, the drugs were suspended in sterilized 0.5% carboxymethylcellulose sodium. **Vitacoenzyme tablets (0.8 g) was purchased from Beihai Sunshine Pharmaceutical Co., Ltd (Guangxi, China, Lot no. 102029), and** Pepsin kit from Nanjing Jiancheng Bioengineering Research Institute (Nanjing, China). MNNG was purchased from TOKYO Chemical Industry Co., Ltd (Tokyo, Japan). Ranitidine hydrochloride capsule was brought from Zhejiang Kangenbei Pharmaceutical Co., Ltd (Zhenjiang, China). TAKARA RNA reverse transcription kits were from Takara Biomedical Technology (Beijing) Co., Ltd (Beijing, China); TOYOBO amplification kit was purchased from toyo textile (Shanghai) biotechnology co., Ltd (Shanghai, China). Primers to amplify the genes ß-actin, Pi3k, Akt1, Mapk11, Fas, Mapk8, caspase3, Mapk14, Tp53, and Vegfα were designed by Shanghai Guanchun Biotechnology Co., Ltd (bioTNT) (Shanghai, China) (Table 1).

**TABLE 1:** Sequence of gene primers.

Gene official symbol	Forward primer sequence	Reverse primer sequence	Product length (bp)
β-Actin	5′ CCT CTA TGC CAA CAC AGT 3′	5′ AGC CAC CAA TCC ACA CAG 3′	155
Pi3k	5′ CAT CAA TGG CAA CAC TCT AAG 3′	5′ AGG ACA GGT GGA TAC GAA AT 3′	97
Akt1	5′ TTC TCA GTG GCA CAA TGT CAG 3′	5′ GGA TGA AGG TGT TGG G 3′	64
Mapk11	5′ CAA CCC TCT GGC TGT AGA CCT 3′	5′ CGC ACT GAC TCT CTG GTC ACT 3′	67
Faslg	5′ TGC CTC CAC TAA GCC CTC TA 3′	5′ CCT AAC CCC ATT CCA ACC AG 3′	100
Mapk8	5′ AGT GAG CAG AGC AGG CAT AGT 3′	5′ CAG GAG CAG CAC CAT TCT TAC 3′	108
Caspase3	5′ ATG TGT GAA CTT GGT TGG CTT 3′	5′ AGA AAC AAA TGC TGG ATC 3′	90
Mapk14	5′ ACA CCC CCT GCT TAT CTC A 3′	5′ AAG TTC ATC TTC GGC ATC TG 3′	89
Tp53	5′ CAT CTT CCG TCC CTT CTC AAA 3′	5′ AGA CTT GGC TGT CCC TGA CTG 3′	83
Vegfα	5′ TTT CGG GAA CTA GAC CTC TCA 3′	5′ TCA GGC TTT CTG GAT TAA GGA 3′	102
Foxo4	5′ GTC TTT GTC AGC AGG AGA AGG 3′	5′ GAG GTG GTG GTG TAT CAG AGG 3′	80

### Ethics Statement

All feeding conditions were in compliance with the Chinese Animal Welfare Law and the relevant regulations of Fudan University and Shanghai University of Traditional Chinese Medicine Experimental Animal Ethics Committee. Wistar rats from the Experimental Animal Center of Shanghai University of Traditional Chinese Medicine were recruited to establish the model of gastric precancerous lesions. Animal license Code: SYXK Shanghai 2014-008. Ethics No.: PZSHUTCM19039006.

### Animal Handling

#### Animal Model for Drug Metabolism Experiment in Rats

Ten specific pathogen-free male Sprague-Dawley rats (200 g) were provided by the Experimental Animal Center of Shanghai University of Traditional Chinese Medicine. Rats were housed in an animal room (24 ± 2°C, 60 ± 5% relative humidity) with the setting of a 12 h dark/12 h light cycle. Before the experiment, rats were given water and fed standard laboratory food for acclimatization for a week. Rats were fasted for 12 h before the sample collection but still had access to water throughout the experiment. WFC (dissolved in purified water) was orally administered to six rats at a dose of 4.0 g/kg (capsule powder/body weight) twice a day consecutively for 7 days to accumulate as many absorbed components as possible. Four rats were assigned to the blank control group. Whole blood samples (0.5 ml) were collected from the sub-orbital vein and placed in heparinized polythene tubes at 15, 30, 60, 90, and 120 min after the last drug administration. Plasma was separated immediately by centrifugation at 12,000 rpm for 10 min at 4°C. All samples were immediately stored at −80°C until further analysis.

#### Pharmacological Experiment of Precancerous Lesions of Gastric Cancer Model in Rats

Fifty 7-week-old male Wistar rats (171 ± 10 g, SPF Grade) were obtained from the Experimental Animal Center of Shanghai University of Traditional Chinese Medicine. All rats were supported and observed in research facility under alternating light and dark conditions (12 h:12 h). After 3 days of adaptive feeding, all rats were divided into five groups, normal group (N group), model group (M group), high-dose group (WH group), low-dose group (WL group), and control group (VM group), stratified randomly according to the body weight of 10 rats in each group. Based on literature modeling methods (Lin et al., 2019) and with the exception of the N group rats, all rats were given 100 mg/L methyl nitroguanidine aqueous solution drink and 0.3 g/L ranitidine fodder each day, and alternatively gavage fed 150 g/L sodium chloride solution at 56°C and 30% ethanol at 10 ml/kg. All model rats were starved and had satiation disorder. After 4 weeks, drugs were administered by gavage. Drug dosage was as follows: Rats in WH group and WL group were given WFC at 1.44 g/kg and 0.72 g/kg, respectively, and VM group was given vatacoenayme at 0.6 g/kg each day. Rats in N group were given gastric gavage of 10 ml/kg of normal saline. After fasting for 12 h at the end of the 16th week, all rats were sacrificed for sample collection.

### Plasma Sample Preparation

All plasma samples from the experimental rats were combined into one sample so as to eliminate the individual variability in each experiment. To get the whole information of absorbed components and metabolites, equivalent plasma was taken from the five time points to form mixture as the analysis plasma. An aliquot of 3 ml of methanol was added to 1 ml of plasma and vortex-mixed for 3 min to precipitate proteins (PPT). After centrifugation at 12,000 rpm for 10 min, the supernatant was transferred into a clean tube and evaporated to dryness under a gentle stream of nitrogen at room temperature. The residue was re-dissolved in 200 μl initial mobile phase through vortex-mixing and ultrasonic processing. The resulting solution was centrifuged at 12,000 rpm for 10 min, and 5 μl of the supernatant was injected into the LC-MS system for analysis. The blank plasma sample was prepared as the drug-containing sample.

### Preparation and Observation of Animal Samples of Precancerous Lesions of Gastric Cancer Model

#### Pepsin Detection and Histopathology Observation

Body weight and autonomic activity of rats were observed. Detection of pepsin was conducted in accordance with the kit instructions of Nanjing Jiancheng biological engineering institute. The gastric tissues of rats were subjected to tissue dehydration and paraffin discharge operations after being fully fixed with formaldehyde. The embedded tissues were cut into 0.04 μm blank tissue slices for HE staining and histomorphologic study of gastric mucosa under microscope (100×). Inflammatory cell infiltration, ulcer formation, glandular atrophy, basement membrane thickening, and dysplasia were observed.

#### Real-Time Polymerase Chain Reaction

According to operating manual, gastric tissues were extracted by “one-step” Trizol method and concentrations were calculated. Obtained RNA templates were reverse-transcribed into c-DNA using TAKARA RNA Reverse Transcription Kit. Next, RT-qPCR was performed following TOYOBO Amplification Kit instructions. Here, each well contained a PCR reaction volume of 10 μl (SYBR Green master mix 5 μl, forward primer 1 μl, reverse primer 1 μl, c-DNA sample 1 μl, and diethylpyrocarbonate 2 μl). ß-Actin was used as an endogenous control gene. PCR reaction consisted of 45 cycles as follows: 50°C for 2 min → 95°C for 1 min → 95°C for 15 s → 60°C for 1 min → 95°C for 15 s → 60°C for 1 min. 2^−∆Ct^ represents the expression level of the tested genes relative to the normal group of genes.

### Ultra-High Performance Liquid Chromatography and Mass Conditions

#### Chromatographic Conditions

UHPLC-MS conditions and data processing were described as previously reported (Wang et al., 2019). Chromatographic separations were performed on an Acquity UHPLC system (Waters, United States) equipped with a binary solvent delivery system and an autosampler. The extracts were separated using an Acquity UHPLC BEH C18 RP column (1.7 μm, 100 mm × 2.1 mm i. d.; Waters, United States) in which the column temperature was maintained at 45°C to avoid excessive column pressure. The autosampler temperature was fixed at 4°C. The mobile phase consisted of 0.1% formic acid in deionized water (mobile phase A) and acetonitrile (mobile phase B). Separation was conducted with the following gradient elution: 0–3.5 min, 5–20% B; 3.5–8.0 min, 20% B; 8.0–15.0 min, 20–30% B; 15.0–21.0 min, 30–50% B; 21.0–26.0 min, 50–95% B; 26.0–28.0 min, 95% B; 28.0–28.1 min, 95–5% B; and 28.1–32.0 min, 5% B for equilibration of the column. The flow rate was set at 0.3 ml/min, and an aliquot of 5 μl was set as injection volume.

#### Mass Spectrometric Conditions

MS detection was performed using Acquity Synapt G2 Q-TOF tandem mass spectrometer (Waters, United Kingdom) connected to the UHPLC system by an ESI interface and controlled by MassLynx version 4.1 (Waters, United Kingdom). The ESI source was operated in both positive (ESI^+^) and negative (ESI^−^) ionization modes. The optimized conditions to trigger maximum response of metabolites were listed as follows: capillary voltage, −2.5 kV (ESI^−^) or +3 kV (ESI^+^); sample cone, −25 V (ESI^−^) or +30 V (ESI^+^); extraction cone, −4.0 V (ESI^−^) or +4.0 V (ESI^+^); source temperature, 120°C; desolation temperature, 350°C; cone gas (nitrogen) flow, 50 L/h; and desolvation gas (nitrogen) flow, 600 L/h. Argon was used as collision gas. Leucine-enkephalin (2 ng/ml) was used as the lock mass generating a reference ion at m/z of 554.2615 (ESI^−^) or 556.2771 (ESI^+^) by a lock spray at 5 μl/min to acquire accurate mass during analysis.

Data were collected in a centroid mode. MSE approach was conducted with two scan functions. In function 1, the following parameters were set: m/z 50–1,500; scan duration, 0.3 s; interscan delay, 0.024 s; and collision energy ramp, 4 V. In function 2, the following parameters were set: m/z 50–1,500; scan duration, 0.3 s; interscan delay, 0.024 s; and collision energy ramp, 10–30 V. In MSE, MS, and MS/MS data can be acquired almost simultaneously in a single analytical run. Data acquisition and processing were conducted using Waters MassLynx version 4.1.

### Data Processing

#### Construction of In-House Library

By comprehensive document retrieval, information about compounds in the three crude medicinal materials and WFC prescription was collected to form an in-house library of WFC. The in-house library content was described with respect to the known global phytochemical constituents and the metabolites database, including the English name, structure, molecular formula, characteristic fragment ions, all accurate monoisotopic mass values of the related chemical formula, and original source.

#### MassLynx Processing Approach for Analysis of Wei-Fu-Chun Phytochemical Constituents, Absorbed Compounds and Metabolites

Mass data processing was carried out using MassLynx 4.1, including extracted ion chromatograms (EIC) using a narrow mass window of 0.01 Da, calculation of EIC with mass errors within 5 ppm, and fraction isotope abundance value of 1.0. In particular, EIC were also applied to distinguish mixed peaks eluted almost at the same retention time. Target chemical structures were analyzed and confirmed based on EIC, accurately measured mass value, fragment behavior, and elution order, which were all compared with the in-house library data. Furthermore, WFC components in drug-containing samples were also determined by comparing the retention time and mass data with blank samples. By comparing postdose rat serum, pre-dose rat serum, and the extracts of WFC by UHPLC-ESI-Q-TOF/MS, absorbed compound profile of rat serum was obtained and analyzed. During the same retention time, peaks appeared in the drug-containing sample and the extracts sample of WFC, but were absent in the control sample or the peaks in the drug-containing sample were five folds greater than that in the control sample, which was extracted as absorption compound. These candidates were further collated and culled by serious MS spectra analysis to conform the true absorbed compounds.

### Active Chemical Compositions of Wei-Fu-Chun and Potential Targets

According to the results of chemical profiling and metabolic study of WFC, we selected absorbed parent molecules and metabolites in rat serum as candidate compounds. The candidate compounds were paired one-to-one with protein targets using Stitch 5.0 database (http://stitch.embl.de/). We set the parameter to “*Homo sapiens*” with confidence greater than 0.4, and deleted candidate compounds without corresponding targets. Active components and corresponding potential targets were obtained.

### Identifying Precancerous Lesion of Gastric Cancer Related Targets in Wei-Fu-Chun

“Precancerous lesion of gastric cancer” was used as a keyword to search Genecards (https://www.genecards.org/) and OMIM database (http://www.omim.org/) separately and to retrieve therapeutic targets for PLGC. Precancerous lesions of gastric cancer -associated target proteins were collected.

### Targets GO-Enrichment and Pathways Enrichment Analysis

The targets of WFC in the treatment of PLGC corresponding to the selected active components were input into DAVID 6.8 database (https://david.ncifcrf.gov/) for GO enrichment analysis, and in KEGG database (http://www.genome.jp/kegg) for pathway enrichment analysis. The parameters were set to *p* < 0.5 and FDR < 0.05.

### Network Construction and Its Features

We input the active component-target pair obtained from String database (http://string-db.org/cgi/input.pl) into Cytoscape 3.1, set the properties of the active components and targets, selected the most suitable node distribution form, and generated the active component-target interaction map. The primary pathway, biological processes, cellular components and molecular functions of *p* < 0.05 were selected. Visualize the network of “origins-components-targets-pathways” with the Merge feature of Cytoscape 3.1.

### Statistical Analysis

**SPSS 21.0 statistical software was used for data** statistics and analysis. Data measurement was expressed as mean ± standard errors (±SEM). One-way ANOVA was used for comparison among groups, and LSD-t test for further two-paired comparison. A *p*-value <0.05 indicated a significant statistical difference between two groups.

## Results

### Sample Acquisition and LC-MS Conditions of Developed Method

Nowadays, UHPLC-ESI-Q-TOF/MS capable of high resolution, high sensitivity and high accuracy have been proven to be an excellent technique for quantitative and qualitative analysis of multi-components and metabolites in complex mixture especially in TCM formulas. Here, UHPLC-ESI-Q-TOF/MS has shown superior performance, in terms of high mass resolution and accurate mass measurement, fast scan speed and wide dynamic range of mass analyzer, as well as eﬃcient separation capability and speed of UHPLC. The acquisition of dependable biological samples for UHPLC-ESI-Q-TOF/MS analysis played a crucial part in the comprehensive *in vivo* screening of WFC compounds. Primarily, all the possible components should be contained in the collected crude samples and have adequate levels to be detected, taken into account that drug administration method and collection time influence sample content (Zhang et al., 2017).

LC-MS conditions were optimized to enable samples to obtain the best instrumental performance. Both positive and negative ion modes were employed to screen as many potential compounds as possible, but the negative ion mode provided higher signal intensity and the ability to detect more peak signals. Different mobile phase systems and gradient programs were emphasized and investigated to achieve good ionization and separation behavior. A mixture of 0.1% aqueous formic acid and acetonitrile was nally chosen as the preferred mobile phase. This was because acetonitrile had stronger eluting power and the retention behavior of flavonoids on the reversed-phase column was easily affected by pH (increasing pH enhanced the ionization of flavonoids and could reduce the retention in a reversed-phase separation). Thus, small amounts of formic acids were normally included in the solvent to suppress ionization of phenolic or carboxylic groups, hence improving the resolution and reproducibility of each separation. Formic acid buffer have been used as part of the mobile phase for optimizing the analysis time and enhancing separation (Wang et al., 2019). Many components, especially flavonoids and ginsenosides, gave prominent molecular adductive ions when formic acid was used. The results indicated that the proposed method was acceptable and adequate for the comprehensive study of WFC.

### Analysis of Wei-Fu-Chun Phytochemistry Components, Absorbed Components, and Metabolites by UHPLC-ESI-Q-TOF/MS

**FIGURE 1:**
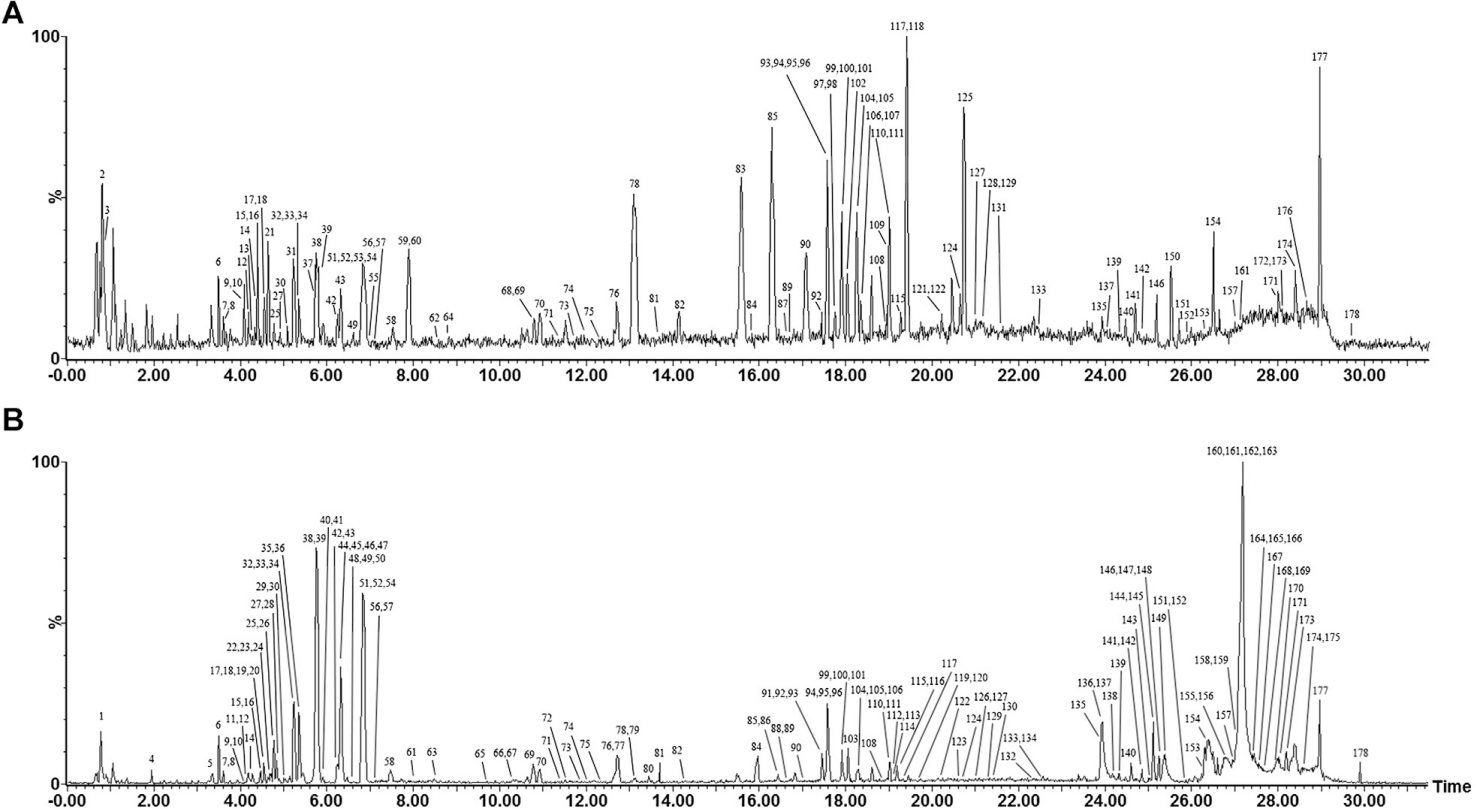
Representative base peak intensity (BPI) chromatograms of WFC in positive mode **(A)** and in negative mode **(B)** and total ions chromatogram (TIC) in positive mode **(C)** and in negative mode **(D)** by UHPLC-ESI-Q-TOF/MS.

The base peak intensity (BPI) chromatograms of WFC extract at negative and positive ion modes are shown in Figure 1 and Table 2. A total of 178 compounds were identified or tentatively characterized, including 70 terpenes (56 diterpenoids, 12 triterpene, and 2 sesquiterpenes), 51 flavonoids, 33 saponin, six phenylpropanoids, four lignans, three coumarins, three organic acids, two fatty acid, one quinones, one sterol, and four unknown compounds. Structurally related compounds shared analogous MS response and fragment behavior. All compounds were characterized based on their ECs, MS data, and retention behavior with the help of the constructed in-house library and additional literature. The library made the workflow significantly more effective when one was familiar with the detailed information of these extensively investigated molecules and allowed the characterization of these compounds even when reference standards were not available. The calculated correct monoisotopic mass values of quasimolecular ions and rational fragment ions were especially critical to screen the various compounds fast and firmly. There were many isomers inevitably existing in the complex natural compounds and the tiny difference of the mass spectra made them diﬃcult to distinguish. In this case, the electronic effect and their hydrophilicity should be considered (Li et al., 2015). The detailed identified information, including chemical formula, observed mass values of quasi-molecular ions, mass error, and botanical source, is listed in Table 2. A chemical library, including 569 compounds in WFC, was established. By matching against the chemical library, 178 ingredients in WFC were identified. Among them, 93 compounds belonged to XCC, 51 to ZQ, 31 to HS. Three compounds were unknown, meaning that the herb(s) from which they came remained undetermined. In addition, 77 absorbed parent molecules and nine metabolites in rat serum were characterized by UHPLC-ESI-Q-TOF/MS.

**TABLE 2:** Characterization of chemical constituents of WFC Tablet by UHPLC-ESI-Q-TOF/MS.

No	RT (min)	Identification	Formula	(M − H)^-^		(M + H)^+^		Other adduct ions in negative mode (−) or positive mode (+)	Structure class	Plant material
				Meas	ppm	Meas	ppm			
1	0.80	Quinic acid	C_7_H_12_O_6_	191.0548	−4.2	—	—	(−)165.0378	Organic acids	ZQ
2	0.88	Kaempferol	C_15_H_10_O_6_	—	—	287.0562	2.1	—	Flavonoids	ZQ
3	0.93	Ginsenoside Re4	C_47_H_80_O_18_	—	—	933.5447	2.6	—	Saponin	HS
4	1.97	Flavogadorinin B	C_23_H_24_O_11_	475.1237	−0.6	—	—	—	Flavonoids	XCC
5*	3.35	Ferulic acid	C_10_H_10_O_4_	193.0496	−2.6	—	—	—	Organic acids	XCC
6*	3.46	Vicenin II	C_27_H_30_O_15_	593.1489	−2.9	595.1667	0.7	(+)577.1447, (+)505.1375	Flavonoids	ZQ
7	3.56	Sucrose	C_12_H_22_O_11_	341.1085	0.3	—	—	—	Saccharide	XCC
8	3.58	Naringenin-7-O-sophorose	C_27_H_32_O_15_	595.1687	4.0	597.1794	−4.2	—	Flavonoids	ZQ
9	4.05	Hubeirubesin B	C_24_H_32_O_6_	415.2126	1.2	—	—	—	Diterpenoids	XCC
10*	4.08	Apigenin-7-O-glucuronide	C_21_H_18_O_11_	445.0791	4.5	447.0951	5.4	—	Flavonoids	XCC
11	4.11	Methyl rosmarinate	C_19_H_18_O_8_	373.0897	−7.0	—	—	—	Phenylpropanoids	XCC
12*	4.14	Vicenin Ⅲ	C_26_H_28_O_14_	563.1383	−3.2	565.1539	v3.2	—	Flavonoids	XCC
13	4.24	Stellarin-2	C_28_H_32_O_16_	—	—	625.1796	4.3	—	Flavonoids	ZQ
14*	4.34	Trichorabdal H	C_22_H_28_O_7_	403.1738	−4.7	405.1921	2.0	—	Diterpenoids	XCC
15*	4.45	Glucosyl-naringin	C_33_H_42_O_19_	741.2271	3.9	743.2407	1.1	(−)609.1470	Flavonoids	ZQ
16	4.47	Rutin	C_27_H_30_O_16_	609.1475	3.1	611.1607	−0.8	(+)303.0536	Flavonoids	XCC
17*	4.53	Naringenin-7-O-triglycoside	C_33_H_42_O_19_	741.2239	−0.4	743.2428	3.9	(−)427.1591	Flavonoids	ZQ
18	4.54	Hyuganoside Ⅱ/hyuganoside Ⅴ	C_20_H_28_O_10_	427.1615	2.6	429.1767	1.4	(−)265.1102	Phenylpropanoids	ZQ
19	4.56	Eriocitrin	C_27_H_32_O_15_	595.1683	3.4	—	—	(−)287.0508	Flavonoids	ZQ
20	4.56	Isovitexin	C_21_H_20_O_10_	431.0994	3.7	—	—	—	Flavonoids	ZQ
21	4.64	Notoginsenoside R1	C_47_H_80_O_18_	—	—	933.5438	1.6	—	Saponin	HS
22	4.64	Epinodosinol	C_20_H_26_O_6_	361.1644	−1.9	—	—	—	Diterpenoids	XCC
23*	4.66	Oreskaurin B	C_22_H_30_O_6_	389.1956	−2.1	—	—	—	Diterpenoids	XCC
24	4.72	Unknown	C_27_H_44_O_11_	543.2820	2.8	—	—	(−)463.0919, 544.2788	—	—
25*	4.78	Neoeriocitrin	C_27_H_32_O_15_	595.1678	2.5	597.1824	0.8	—	Flavonoids	ZQ
26*	4.79	Limonin-17-β-D-glucopyranoside	C_32_H_42_O_14_	649.2494	−0.3	—	—	—	Triterpene	ZQ
27*	4.86	3′-Methoxyl isovitexin	C_22_H_22_O_11_	461.1084	0	463.1232	−1.7	(+)427.1087	Flavonoids	ZQ
28	4.87	Henryin	C_22_H_32_O_6_	391.2112	−2.3	—	—	—	Diterpenoids	XCC
29	5.06	Melissoidesin K	C_24_H_36_O_8_	451.2341	2	—	—	—	Diterpenoids	XCC
30*	5.08	(+)-Rabdosiin or (−)-rabdosiin	C_36_H_30_O_16_	717.1461	0.7	719.1617	0.7	(−)519.0913	Phenylpropanoids	XCC
31*	5.17	Loquatoside	C_20_H_22_O_11_	—	—	439.1230	−2.3	—	Flavonoids	ZQ
32	5.35	Narirutin	C_27_H_32_O_14_	579.1713	−0.2	581.1866	−0.7	(+)435.1089, (+)273.0668	Flavonoids	ZQ
33*	5.37	Kamebacetal A	C_21_H_30_O_5_	361.2004	−3.0	363.2163	−2.2	—	Diterpenoids	XCC
34*	5.38	(+)-Rabdosiin or (−)-rabdosiin	C_36_H_30_O_16_	717.1461	0.7	719.1617	0.7	(−)519.0913	Phenylpropanoids	XCC
35	5.40	Narirutin-4′-glucoside	C_33_H_42_O_19_	741.2217	−3.4	—	—	—	Flavonoids	ZQ
36	5.43	Miscanthoside	C_21_H_22_O_11_	449.1080	−0.9	—	—	—	Flavonoids	ZQ
37	5.70	Rhoifolin	C_27_H_30_O_14_	—	—	579.1710	−0.7	(+)273.0814	Flavonoids	ZQ
38*	5.76	Naringin	C_27_H_32_O_14_	579.1739	4.3	581.1870	0	(+)273.0774	Flavonoids	ZQ
39*	5.83	Vitexin	C_21_H_20_O_10_	431.0961	−3.9	433.1144	2.1	—	Flavonoids	XCC
40	5.91	Naringenin	C_15_H_12_O_5_	271.0606	0	—	—	—	Flavonoids	ZQ
41	5.91	Enmenol	C_20_H_30_O_6_	365.1971	1.9	—	—	—	Diterpenoids	XCC
42*	6.25	Hesperidin	C_28_H_34_O_15_	609.1844	4.1	611.1957	−3.1	(+)449.1443, (+) 303.0813	Flavonoids	ZQ
43*	6.3	Caffeic acid	C_9_H_8_O_4_	179.0339	−2.8	181.0504	1.7	—	Organic acids	XCC
44	6.32	Ladanein	C_17_H_14_O_6_	313.071	−0.6	—	—	—	Flavonoids	XCC
45	6.32	Rosmarinic acid	C_18_H_16_O_8_	359.0778	3.1	—	—	—	Phenylpropanoids	XCC
46	6.32	Cirsiliol	C_17_H_14_O_7_	329.065	−3.3	—	—	—	Flavonoids	XCC
47	6.33	Danshensu	C_9_H_10_O_5_	197.0445	−2.5	—	—	—	Phenylpropanoids	XCC
48*	6.60	Lushanrubescensin F	C_21_H_32_O_7_	395.207	0	—	—	—	Diterpenoids	XCC
49	6.63	THDO	C_20_H_28_O_4_	331.1912	0.9	333.2078	3.6	—	Diterpenoids	XCC
50	6.72	(+)-1-Hydroxypinoresinol	C_20_H_22_O_7_	373.1288	0.3	—	—	—	Lignans	XCC
51*	6.81	Lariciresinol	C_20_H_24_O_6_	359.1479	−4.5	361.1634	−4.7	—	Lignans	XCC
52	6.83	Neohesperidin	C_28_H_34_O_15_	609.1829	1.6	611.1970	−1.0	(+)449.1426, (+)303.0884	Flavonoids	ZQ
53	6.83	Hesperetin-5-O-glucoside	C_22_H_24_O_11_	—	—	465.1381	−3.4	—	Flavonoids	ZQ
54*	6.86	Hesperetin	C_16_H_14_O_6_	301.0704	−2.7	303.0874	1.6	—	Flavonoids	ZQ
55*	6.92	LHMG	C_29_H_32_O_17_	—	—	653.1722	0.6	(+)347.0763	Flavonoids	ZQ
56*	7.08	Nomilinic acid glucoside	C_34_H_48_O_16_	711.2845	−2.7	713.3016	−0.7	—	Triterpene	ZQ
57*	7.14	Hesperetin-7-O-glucoside	C_22_H_24_O_11_	463.1247	1.5	465.1413	3.4	—	Flavonoids	ZQ
58	7.48	Pedalitin	C_16_H_12_O_7_	315.0520	4.8	317.0670	2.8	—	Flavonoids	XCC
59*	7.90	Sudachinoid A	C_26_H_34_O_9_	—	—	491.2298	3.5	—	Sesquiterpenes	ZQ
60	7.90	Meranzin	C_15_H_16_O_4_	—	—	261.1139	4.6	(−)189.0572	Coumarins	ZQ
61	8.00	Deacetyl nomilin glucoside	C_34_H_46_O_15_	693.2727	−4.5	—	—	—	Triterpene	ZQ
62*	8.24	7-O-6″-Malonylnaringin	C_30_H_34_O_17_	—	—	667.1877	0.4	—	Flavonoids	ZQ
63*	8.49	Lasiodonin	C_20_H_28_O_6_	363.1815	1.9	—	—	—	Diterpenoids	XCC
64	8.81	Hebeirubescensin H or G	C_20_H_28_O_7_	—	—	381.19	−3.4	—	Diterpenoids	XCC
65	9.78	Effusanin A	C_20_H_28_O_5_	347.1855	−0.9	—	—	—	Diterpenoids	XCC
66	10.26	Sesamin	C_20_H_18_O_6_	353.103	1.4	—	—	—	Lignans	XCC
67	10.33	Obacunone 17-O-β-D-glucoside	C_32_H_42_O_13_	633.2539	−1.3	—	—	—	Triterpenes	ZQ
68	10.78	Ginsenoside Rg1	C_42_H_72_O_14_	—	—	801.4998	−0.2	(−)845.4928 [M + HCOO]^−^	Saponin	HS
69	10.79	Schaftoside	C_26_H_28_O_14_	563.1389	−2.1	565.158	4.1	—	Flavonoids	XCC
70	10.93	Ginsenoside Re	C_48_H_82_O_18_	945.5353	2.6	947.5560	−2.0	(−)991.5504 [M + HCOO]^−^	Saponin	HS
71*	11.40	Excisanin A	C_20_H_30_O_5_	349.2021	1.7	351.2193	6.3	—	Flavonoids	XCC
72	11.52	Rabdosinate/Gesneroidin C	C_28_H_38_O_10_	533.2367	−3.8	—	—	—	Diterpenoids	XCC
73*	11.72	Melissoidesin T	C_24_H_36_O_7_	435.2396	3.0	437.2491	−11	—	Diterpenoids	XCC
74	11.99	Neoponcirin	C_28_H_34_O_14_	593.1849	−3.5	595.2009	−3.0	—	Flavonoids	ZQ
75*	12.36	Sodoponin	C_22_H_32_O_7_	407.2076	1.5	409.2215	−2.7	(−)285.0743	Diterpenoids	XCC
76	12.64	Poncirin	C_28_H_34_O_14_	593.1874	0.7	595.2017	−1.7	—	Flavonoids	ZQ
77*	12.67	Rabdophyllin H	C_24_H_36_O_9_	467.2281	0	—	—	—	Diterpenoids	XCC
78*	13.09	Glaucocalyxin A	C_20_H_28_O_4_	331.1921	3.6	333.2067	0.3	—	Diterpenoids	XCC
79	13.14	Lasiokaurin	C_22_H_30_O_7_	405.1931	4.4	—	—	—	Diterpenoids	XCC
80*	13.43	Oreskaurin C	C_20_H_30_O_5_	349.2020	1.4	—	—	—	Diterpenoids	XCC
81*	13.73	Oridonin	C_20_H_28_O_6_	363.1826	5.0	365.1955	−2.5	—	Diterpenoids	XCC
82*	14.27	Fumotonaringin	C_28_H_34_O_14_	593.1876	1.0	595.2047	3.4	—	Flavonoids	ZQ
83	15.67	NHMG	C_33_H_40_O_18_	—	—	725.2277	−2.2	—	Flavonoids	ZQ
84	15.92	Coetsoidin A	C_22_H_26_O_6_	385.1665	3.6	387.1806	−0.5	—	Diterpenoids	XCC
85*	16.43	Ginsenoside Rf	C_42_H_72_O_14_	799.4832	5.8	801.4992	−1.0	(−)845.4948 [M + HCOO]^−^	Saponin	HS
86	16.54	Melissoidesin N	C_22_H_32_O_5_	375.2185	3.7	—	—	—	Diterpenoids	XCC
87	16.60	Epoxybergamottin	C_21_H_22_O_5_	—	—	355.1542	−0.8	—	Coumarins	ZQ
88	16.61	Melissoidesin P	C_22_H_34_O_6_	393.2292	3.8	—	—	—	Diterpenoids	XCC
89*	16.65	Isosinensetin	C_20_H_20_O_7_	371.1142	3.0	373.1283	−1.1	—	Flavonoids	ZQ
90*	17.03	20(S) or 20(R)-Notoginsenoside R2	C_41_H_70_O_13_	769.4771	2.0	771.4866	−3.8	(−)815.4809 [M + HCOO]^−^	Saponin	HS
91	17.44	Ent-abierubesin A	C_20_H_32_O_5_	351.2173	0.6	—	—	—	Diterpenoids	XCC
92*	17.46	Ginsenoside Ra2	C_58_H_98_O_26_	1,209.6309	3.4	1,211.6406	−1.6	(−)1,255.6528 [M + HCOO]^−^	Saponin	HS
93*	17.53	Melissoidesin U	C_26_H_38_O_8_	477.2478	−2.1	479.2637	−1.7	—	Diterpenoids	XCC
94	17.59	20(S)-Ginsenoside Rd	C_48_H_82_O_18_	945.5407	−1.7	947.5563	−1.7	—	Saponin	HS
95*	17.62	Ginsenoside Rb_1_	C_54_H_92_O_23_	1,107.5985	3.1	1,109.6107	−0.1	(−)1,153.6080 [M + HCOO]^−^	Saponin	HS
96*	17.62	20(S)-Ginsenoside Rg2	C_42_H_72_O_13_	783.4938	4.6	785.5085	4.3	(−)829.4987, 621.3106	Saponin	HS
97	17.75	Malonyl ginsenoside Rb_1_	C_57_H_94_O_26_	—	—	1,195.6149	3.1	—	Saponin	HS
98*	17.78	Ginsenoside Ra3/nootoginsenoside Fa	C_59_H_100_O_27_	—	—	1,241.6506	−1.9	—	Saponin	HS
99*	17.86	Ginsenoside Rb2	C_53_H_90_O_22_	1,077.5875	−0.8	1,079.5979	−2.1	(−)1,123.5891 [M + HCOO]^−^	Saponin	HS
100*	17.92	Ginsenoside Rb3/ginsenoside Rc	C_53_H_90_O_22_	1,077.5875	1.2	1,079.5975	−2.5	(−)1,123.5913 [M + HCOO]^−^	Saponin	HS
101	17.94	Ginsenoside Ra1	C_58_H_98_O_26_	1,209.6241	−1.3	1,077.5929	−1.8	(−)1,255.6307 [M + HCOO]^−^	Saponin	HS
102	18.04	Auranetin	C_20_H_20_O_7_	—	—	373.1275	−3.2	—	Flavonoids	ZQ
103*	18.07	Ginsenoside Ro	C_48_H_76_O_19_	955.4911	0.8	—	—	—	Saponin	HS
104*	18.24	5-Demethylnobiletin	C_20_H_20_O_8_	387.1078	−0.5	389.122	−4.1	—	Flavonoids	ZQ
105	18.25	Limonin	C_26_H_30_O_8_	469.1869	1.5	471.2009	−2.1	—	Triterpene	ZQ
106*	18.30	Malonyl-notoginsenoside R4	C_62_H_102_O_30_	1,325.6366	−0.9	1,327.6525	−0.7	—	Saponin	HS
107	18.33	Tetramethyl-O-isoscutellarein	C_19_H_18_O_6_	—	—	343.118	−0.6	—	Flavonoids	ZQ
108*	18.85	Pomiferin F	C_20_H_28_O_3_	315.1955	−1.6	317.2102	−4.7	—	Diterpenoids	XCC
109	18.95	Ginsenoside Rs1	C_55_H_92_O_23_	—	—	1,121.6149	3.7	—	Saponin	HS
110	18.97	Ginsenoside Rs2	C_55_H_92_O_23_	1,119.6171	−0.8	1,121.6077	−2.8	(−)1,165.5997 [M + HCOO]^−^	Saponin	HS
111*	18.99	Quinquenoside R1	C_56_H_94_O_24_	1,149.6064	0.6	1,151.6240	2.3	—	Saponin	HS
112*	19.02	20(R)-Ginsenoside Rd	C_48_H_82_O_18_	945.5430	0.1	—	—	(−)991.5479, 621.7865	Saponin	HS
113*	19.05	Malonyl-ginsenoside Re	C_51_H_84_O_21_	1,031.5408	−1.8	1,033.5574	−0.9	—	Saponin	HS
114	19.12	Obacunoic acid	C_26_H_32_O_8_	471.2017	−0.4	—	—	—	Triterpene	ZQ
115*	19.20	Nomilinic acid	C_28_H_36_O_10_	531.2222	−1.5	533.2375	−2.3	(−)427.2114	Triterpene	ZQ
116	19.23	Shikokianin	C_24_H_32_O_8_	447.2003	−3.6	—	—	—	Diterpenoids	XCC
117*	19.30	Angustifolin	C_21_H_28_O_6_	375.1806	−0.5	377.1962	−0.5	—	Triterpene	XCC
118	19.41	Gossypetin hexamethyl ether	C_21_H_22_O_8_	—	—	403.1395	0.5	(+)373.0964	Flavonoids	ZQ
119	19.68	Melissoidesin L	C_22_H_32_O_4_	359.2222	0	—	—	—	Diterpenoids	XCC
120	19.76	β-Sitosterol	C_29_H_50_O	413.3776	−1.7	—	—	—	Triterpene	XCC
121	20.22	3-Methoxynobiletin	C_22_H_24_O_9_	—	—	433.1502	0.7	—	Flavonoids	ZQ
122*	20.24	Rabdosichuanin D	C_24_H_34_O_8_	449.2192	3.8	451.2311	−4.7	—	Diterpenoids	XCC
123	20.60	Melissoidesin Q	C_24_H_36_O_7_	435.2393	2.3	—	—	—	Diterpenoids	XCC
124*	20.66	20(R)-Acetyl ginsenoside Rd	C_50_H_84_O_19_	987.5709	−0.4	989.5724	3.9	(−)1,033.5579 [M + HCOO]^−^	Saponin	HS
125*	20.73	Tangeretin/pentamethoxyflavone	C_20_H_20_O_7_	—	—	373.1298	2.9	(+)343.0821	Flavonoids	ZQ
126	20.98	Glaucocalyxin D？	C_22_H_30_O_5_	373.2028	3.5	—	—	—	Diterpenoids	XCC
127	21.03	Ginsenoside Rg6	C_42_H_70_O_12_	765.4799	1.3	767.4957	1.4	(−)811.4885, 459.3017	Saponin	HS
128	21.26	Malonyl-ginsenoside Ra2/Ra1	C_61_H_100_O_29_	—	—	1,297.6469	3.1	—	Saponin	HS
129*	21.31	Ginsenoside Rk1	C_42_H_70_O_12_	765.4709	2.6	767.4954	1.0	(−)811.4865, 603.1678	Saponin	HS
130	21.40	Leukamenin E	C_22_H_32_O_5_	375.2185	3.7	—	—	—	Diterpenoids	XCC
131	21.54	7HPF	C_20_H_20_O_8_	—	—	389.1248	3.1	—	Flavonoids	ZQ
132*	22.35	20(S)-Ginsenoside Rg3	C_42_H_72_O_13_	783.4906	2.3	—	—	(−)829.4968, 621.3106	Saponin	HS
133*	22.46	Unknown	C_44_H_52_O_4_	643.3774	−2.0	645.3930	−2.2	—	—	—
134*	22.57	Glaucocalyxin B	C_22_H_30_O_5_	373.2024	2.4	—	—	—	Diterpenoids	XCC
135	23.97	Ginsenoside F4	C_42_H_70_O_12_	765.4761	4.1	767.4953	0.9	(−)811.4877, 459.8965	Saponin	HS
136	24.06	Ent-abierubesin B	C_20_H_34_O_6_	369.2267	−2.7	—	—	—	Diterpenoids	XCC
137	24.09	Ginsenoside Rg5	C_42_H_70_O_12_	765.4846	−1.0	767.4927	−2.5	(−)811.4836, 603.1898	Saponin	HS
138	24.17	Rabdosin B	C_24_H_32_O_8_	447.2024	1.1	—	—	—	Diterpenoids	XCC
139	24.32	Corosolic Acid	C_30_H_48_O_4_	471.3470	−0.8	473.3618	−2.7	—	Triterpene	XCC
140	24.58	Hexamethoxyflavone（Nobiletin）	C_21_H_22_O_8_	401.1217	−4.7	403.1392	−0.2	(−)371.2429, 315.2524, 239.1493	Flavonoids	ZQ
141	24.82	Melissoidesin R	C_26_H_38_O_8_	477.2472	−3.4	479.2633	−2.5	—	Diterpenoids	XCC
142*	24.88	Lophanic acid	C_20_H_32_O_3_	319.2267	−1.9	321.2433	0.9	—	Diterpenoids	XCC
143	25.00	Melissoidesin I	C_22_H_34_O_6_	393.2289	3.1	—	—	—	Diterpenoids	XCC
144	25.13	Naringenin-7-O-glucoside（Prunin）	C_21_H_22_O_10_	433.1133	−0.5	—	—	—	Flavonoids	ZQ
145*	25.13	Melissoidesin M	C_22_H_34_O_5_	377.2318	−2.7	—	—	—	Diterpenoids	XCC
146*	25.33	Isoscoparin C	C_20_H_32_O_3_	319.2278	1.6	321.2443	4.0	—	Diterpenoids	XCC
147	25.35	Rabdosin E	C_20_H_26_O_7_	377.1603	0.8	—	—	—	Diterpenoids	XCC
148	25.38	Melissoidesin J	C_24_H_36_O_7_	435.2365	−4.1	—	—	—	Diterpenoids	XCC
149	25.46	Eriocalyxin A/Eriocalyxin B	C_20_H_24_O_5_	343.1551	1.7	—	—	—	Diterpenoids	XCC
150*	25.70	7alpha-Hydroxystigmasterol	C_29_H_48_O_2_	—	—	429.3711	−5.1	—	Sterol	XCC
151*	25.89	Unknown	C_20_H_24_O_5_	343.1546	0.3	345.1701	−0.3	—	—	—
152*	25.94	Oreskaurin A	C_22_H_28_O_8_	419.1705	−0.2	421.1868	1.4	—	Diterpenoids	XCC
153	26.32	Dipropyl octadecanedioate	C_24_H_46_O_4_	397.3301	−4.3	399.3456	−4.5	—	Fatty acid	XCC
154	26.43	Taibaijaponicain A	C_21_H_30_O_7_	393.1909	−1.0	395.2078	2.0	—	Diterpenoids	XCC
155	26.82	Gossypetin Hexamethyl Ether	C_21_H_22_O_8_	401.1217	−4.7	—	—	—	Flavonoids	ZQ
156	26.85	Friedelin	C_30_H_50_O	425.3775	−1.9	—	—	—	Saponin	XCC
157*	27.00	Ent-abierubesin D/ent-Abierubesin C	C_20_H_32_O_4_	335.2213	−2.7	337.2386	2.1	—	Diterpenoids	XCC
158*	27.11	Rabdolasional	C_22_H_30_O_7_	405.193	4.2	—	—	—	Diterpenoids	XCC
159	27.11	Isoschaftoside	C_26_H_28_O_14_	563.1411	1.8	—	—	—	Flavonoids	XCC
160	27.17	Hebeirubescensin K	C_20_H_30_O_5_	349.2000	−4.3	—	—	—	Diterpenoids	XCC
161*	27.18	Melissoidesin O	C_24_H_34_O_6_	417.2261	−3.8	419.2418	−3.8	—	Diterpenoids	XCC
162	27.18	Epipinoresinol	C_20_H_22_O_6_	357.1342	1.1	—	—	—	Lignans	XCC
163	27.20	Micranthin C	C_20_H_28_O_5_	347.1853	−1.4	—	—	—	Diterpenoids	XCC
164	27.26	2,6-Dimethoxybenzoquinone	C_8_H_8_O_4_	167.0337	−4.2	—	—	—	Quinones	XCC
165*	27.33	Acetylursolic Acid	C_32_H_50_O_4_	497.3641	2.0	—	—	—	Triterpene	XCC
166	27.34	Tetracosylferulate	C_34_H_58_O_4_	529.4261	0.8	—	—	—	Phenolic acids	XCC
167	27.45	Esculetin	C_9_H_6_O_4_	177.0184	−2.3	—	—	—	Coumarins	XCC
168	27.60	Rabdoinflexin B/rabdokunmin C	C_20_H_30_O_5_	349.2025	2.9	—	—	—	Diterpenoids	XCC
169	27.66	Teuclatriol	C_15_H_28_O_3_	255.1954	−2.4	—	—	—	Sesquiterpenes	XCC
170	27.91	Isoscoparin A	C_22_H_34_O_4_	361.2379	0	—	—	—	Diterpenoids	XCC
171*	28.02	Dibutyl terephthalate	C_16_H_22_O_4_	277.1427	−4.7	279.1601	1.8	—	Fatty acid	XCC
172*	28.15	Notoginsenoside Fe	C_47_H_80_O_17_	—	—	917.5495	2.3	—	Triterpene	HS
173*	28.20	Isoscoparin B	C_21_H_36_O_4_	351.2531	−1.1	353.2681	−3.1	—	Diterpenoids	XCC
174*	28.60	Daucosterol	C_35_H_60_O_6_	575.4319	1.2	577.4431	−6.4	—	Triterpene	XCC
175	28.69	Forrestin B	C_24_H_36_O_8_	451.2318	−3.1	—	—	—	Diterpenoids	XCC
176	28.77	Vinaginsenoside R16	C_47_H_80_O_17_	—	—	917.5478	0.4	—	Saponin	HS
177	28.88	Maoyecrystal L	C_24_H_34_O_8_	449.2169	−1.3	451.2327	−1.1	—	Diterpenoids	XCC
178*	29.67	Quercetin	C_15_H_10_O_7_	301.0359	3.7	303.0502	−1.0	—	Flavonoids	XCC

In the identification column, the compounds marked with * are the components absorbed into blood circulation. In the other adduct ions column, ions (+) were detected in positive mode, ions (−) were detected in negative mode. For the plant material column: HS, Radix Ginseng Rubra; ZQ, *Fructus aurantii*; XCC, *Isodon amethystoides*; 49: THDO, 7α,10α,14β-10,14,18-trihydroxykaura-11,16-dien-15-one; 55: LHMG, limocitrin-3-O-(3-hydroxy-3-methylglutarate)-glucoside; 83: NHMG, natsudaidain-3-O-(3-hydroxy-3-methylglutarate)-glucoside; 131: 7HPF, 7-hydroxyl-4′,3,5,6,8-pentamethoxy-flavone.

#### Identification of Diterpenoids, Triterpene, and Sesquiterpenes

Diterpenoids are the major bioactive constituents of XCC. About 500 new diterpenoids (mainly ent-kauranoids) with different oxygenations and cleavage patterns have been isolated and characterized from plants of the genus Isodon. In our study, 70 terpenes, including 56 diterpenoids, 12 triterpene, and two sesquiterpenes, were detected and identified in WFC, by means of UHPLC-ESI-Q-TOF/MS in both negative and positive mode according to the literatures (Zhou et al., 2009; Jin et al., 2010). The accurate mass measurements and elemental compositions of molecular ions and main product ion were shown in Table 2. In this study, all terpenes from XCC were ionized as deprotonated molecules [M − H]^−^ in negative mode, however, only part of the terpenes were detected in positive mode.

#### Identification of Flavonoids

Fifty-one flavonoids and their glycosides in WFC (39 from ZQ, 12 from XCC) were detected according to the literature (Fabre et al., 2001; Shi et al., 2007; Zhang et al., 2012), most of them having a common structure of C6-C3-C6. Their cleavage regularity in extracts has been well described. Simply, for aglycones, the main MS/MS behavior involved RDA fragmentation pathway and losses of small neutral molecules and radicals from [M − H]^−^, CO (28 Da), CO_2_ (44 Da), H_2_O (18 Da), and CH_3_ (15 Da) that are useful for determining the presence of specific functional groups; for flavonoid glycosides, the cleavage at the glycosideic linkages in positive and negative ion mode both could happen and produce the same fragmentations with low m/z as the fragmentations obtained in their aglycone. For example, flavone glycosides were characterized by the successive losses of an apiose residue C_5_H_8_O_4_ (132 Da), pentose residue (146 Da), hexose residue (162 Da), glucuronic acid (176 Da), rutinoside or glycoside neohesperidin (308 Da).

Compound **15** showed [M − H]^−^ ion at m/z 741, and identical fragmentations at m/z 609, which lost an apiose residue (132 Da). Here, compound **15** was identified as Glucosyl-naringin (Table 2). Compound **18** showed [M − H]^−^ ion at m/z 427, and identical fragmentations at m/z 265, which lost a hexose residue (162 Da). Compound **18** was identified as hyuganoside II or V. Compounds **19**, **32**, and **38** showed [M − H]^−^ ion at m/z 595, 579, 579, and identical fragmentations at m/z 287, 271, 271, which lost a hexose residue (308 Da). Compound **19** was identified as eriocitrin. Both compounds **32** and **38** showed [M − H]^−^ ion at m/z 579 [M + H]^+^ ion at m/z 581, and identical fragmentations at m/z 271 (negative) and 273 (positive), which lost a hexose residue (308 Da), as well as identical fragmentations at m/z 435 (positive), which lost a pentose residue (146 Da) of **38**. Thus, **32** and **38** were deemed narirutin and naringin based on the fragmentations and appearance time. Compounds **42** and **52** were identified as hesperidin and neohesperidin, as both lost a hexose residue (308 Da) at negative and positive conditions (Li et al., 2004). Compound **57** showed identical [M − H]^−^ ion at m/z 463, and fragmentation at m/z 301 and 286, suggesting that compound **57** had a hexose residue (162 Da), and CH_3_ (15 Da) in its aglycone. Compound **57** was identified as hesperetin-7-O-glucoside. Other flavonoids were tentatively identified based on the positive and negative parent ion and previous literature report.

#### Identification of Saponin

Thirty-two saponins (31 from HS) were detected and identified in WFC, by matching the empirical molecular formula and fragment ions with reported MS data of saponins in HS, ZQ, and XCC (Liu et al., 2006; Dan et al., 2009; Zhang et al., 2012). The detailed information was shown in Table 2. Most ginsenosides were liable to form [M − H]^−^ ion and [M + HCOO]^−^ ion in the negative mode and [M + H]^+^ ion in positive mode. The main pathway for mass spectrometry cleavage of ginsenosides to lose the glycosyl groups to form the parent ion of m/z 459 (diol type) and m/z 475 (triol type). For example, in the case of compounds **127** and **135**, m/z **459** was detected in negative mode. The second point is the characteristic fragment ion formed by glycosylation. For compounds **96**, **132**, and **112**, the same fragment m/z 621 lost one or two glucoses from parent ion. Fragment m/z 603 of compounds **129** and **137** lost one glucose from parent ion.

#### Identification of Phenylpropanoids

Six phenylpropanoids were detected in WFC. They were methyl rosmarinate (11), hyuganoside II/hyuganoside V (18) (+)-rabdosiin or (−)-rabdosiin (30, 34), rosmarinic acid (45), danshensu (47) (Zhang et al., 2012). Compound **18** showed [M − H]^−^ ion at m/z 427.1615, and identical fragmentations at m/z 265, which lost a glucose residue (162 Da).

#### Identification of Lignans

Four lignans were detected in WFC, which were (+)-1-hydroxypinoresinol (50), Lariciresinol (51), sesamin (66), and Epipinoresinol (162). All of the four compounds were from XCC. They were liable to form [M − H]^−^ ion in the negative mode and [M + H]^+^ ion in positive mode.

#### Identification of Coumarins

Meranzin (60) and Epoxybergamottin (87) were identified from ZQ, and Esculetin (86) was identified from XCC. Meranzin (60) showed a protonated molecule at m/z 261.1139 [M + H]^+^, with a molecular formula C_15_H_16_O_4_. MS/MS fragmentation appeared in m/z 189.0572 [M + H-C_4_H_7_O]^+^ (Tsujimoto et al., 2019). The MS fragmentation was typical for the fragmentation pattern of meranzin.

#### Identification of Organic Acids, Fatty Acid, Quinones, Sterol, and Unknown Compounds

Three organic acid, two fatty acid, one quinones, one sterol, and four unknown compounds were identified.

#### UHPLC-ESI-Q-TOF/MS Analysis of Compounds in Wei-Fu-Chun Absorbed into Blood Circulation

**FIGURE 2:**
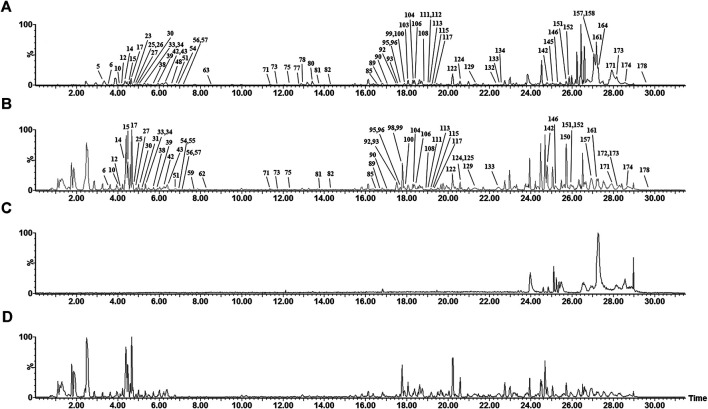
Representative base peak intensity (BPI) chromatograms of absorbed compounds after oral administration of WFC in negative mode **(A)** and in positive mode **(B)** by UHPLC-ESI-Q-TOF/MS; Representative base peak intensity (BPI) chromatograms of blank serum in negative mode **(C)** and in positive mode **(D)** by UHPLC-ESI-Q-TOF/MS.

The absorbed compounds were explored based on the hypothesis that the active constituents were those absorbed into tissue (Zhao et al., 2010). By comparing post-dose rat serum, pre-dose rat serum, and the extracts of WFC by UHPLC-ESI-Q-TOF/MS, an absorbed compounds profile of rat serum was obtained and analyzed. The 77 parent molecules in the positive- and negative-ion mode were summarized in Figure 2 and Table 2 and marked with an asterisk (*). Of these 77 parent molecules, there were 24 diterpenoids (23 from XCC, one from ZQ), 20 flavonoids (15 from ZQ, five from XCC), 17 saponins (all are ginsenoside from HS), five triterpenes (three from XCC, two from ZQ), two phenylpropanoids (both are from XCC), two organic acids (both are from XCC), one sesquiterpenes (from ZQ), one sterol (from XCC), one fatty acid (from XCC), and two unknown compounds. Taken together, the most absorbed compounds were terpene from XCC, flavonoids from ZQ, and saponins from HS.

#### Tentative Identification of the Wei-Fu-Chun Metabolites in Rats

Some drug components could be further metabolized by a variety of metabolic enzymes in the body and form phase I or phase II metabolites, which are highly related to the curative effect and drug elimination. Drug metabolites usually kept the core structure of the parent drug after biotransformation, hence the obtained candidate metabolites were further identified by comparing the change in molecular mass (△M), the retention time, and MS_2_ spectral with their parent drugs. Metabolites of all 178 compounds were tentatively predicted in rat plasma according to their molecular weights and literature reports; however, only the metabolites of limonin, ginsenoside Rg1, ginsenoside Rb3, oridonin were found in our study. M1 eluted at 1.07 min was observed at m/z 455.1891, which is 16 Da (−O) lower than limonin. M2 eluted at 1.08 min and the positive ion was observed at m/z 485.1989, which was 14 Da (+CH_2_) higher than limonin. The peak elution of M4 was at 4.34 min and the positive ion was observed at m/z 489.2314, which was 18 Da (A-ring lactone) higher than that of the parent drug limonin. The two metabolites (M3 and M5) of ginsenoside Rg1 were observed at m/z 859.4590 and 861.4748 in negative mode, which were 14 Da (+O-2H) and 16 Da (+O) higher than limonin (Wang et al., 2016). Oridonin + OH (M9) and +2OH (M8) compounds were observed at m/z 379.1540 and 395.1656.

### Network Pharmacology Approach to Predict the Compounds and Action Mechanisms of Wei-Fu-Chun Against Precancerous Lesions of Gastric Cancer

#### Identification of Active Chemical Compositions, Candidate Targets and Biological Processes for Wei-Fu-Chun Against Precancerous Lesions of Gastric Cancer

The efficacy of TCM is based on the overall regulation of multiple active ingredients acting on different disease-related targets through multiple pathways (Li and Zhang, 2013). Therefore, we collected 86 candidate compounds (Tables 2, 3) and 105 potential targets. 13 active components and 48 protein targets to treat PLGC were then obtained. Detailed information of these therapeutic targets was described in Tables 4, 5 separately.

**TABLE 3:** UHPLC-ESI-Q-TOF/MS data obtained in negative and positive ion detection mode for identification of WFC metabolites.

Number	RT (min)	Identification	Formula	Theoretical mass (m/z)	Experimental mass (m/z) (+)/(−)	Error (ppm)	Fragment ions	Possible original compound
M1	1.07	Limonin-O	C_26_H_30_O_7_	455.2070	455.2051 (+)	−1.9	315.1348, 409.1765	Limonin
M2	1.08	Limonin + CH2	C_27_H_34_O_8_	485.2175	485.2159 (+)	−1.6	453.1816, 187.1044	Limonin
M3	4.33	Ginsenoside (Rg1+O-2H)	C_42_H_70_O_15_	859.4697	859.4690	−0.1	651.2668, 633.2964	Ginsenoside Rg1
M4	4.34	Limonoate A-ring lactone	C_26_H_32_O_9_	489.2125	489.2122 (+)	−0.3	421.2219, 95.0884	Limonin
M5	4.386	Ginsenoside (Rg1+O)	C_42_H_72_O_15_	861.4853	861.4748	−2.0	—	Ginsenoside Rg1
M6	21.29	Ginsenoside CK	C_36_H_62_O_8_	621.4366	621.4383	1.7	459.2689, 160.9794	Ginsenoside Rb3
M7	23.22	Ginsenoside M2′	C_41_H_70_O_12_	753.4789	753.4826	3.7	799.4698, 293.1731	Ginsenoside Rb3
M8	23.89	Oridonin+2OH	C_20_H_28_O_8_	395.1706	395.1696	−1.0	347.2173, 329.1415	Oridonin
M9	26.59	Oridonin + OH	C_20_H_28_O_7_	379.1757	379.1740	−1.7	361.1645, 349.2100, 325.1804, 299.2557	Oridonin

M1, M2, and M4 were detected in positive mode. The rest was detected in negative mode.

**TABLE 4:** Representative active compounds in WFC.

ID	Compound name	Molecular formula	Molecular weight	Plant material	Classification
C1	Caffeic acid	C_9_H_8_O_4_	180.159	HS	Organic acids
C2	Ginsenoside Rf	C_42_H_72_O_14_	801.024	HS	Saponin
C3	Ginsenoside Rb_1_	C_54_H_92_O_23_	1,109.307	HS	Saponin
C4	Tangeretin	C_20_H_20_O_7_	372.373	HS	Flavonoids
C5	Ginsenoside Rg_1_	C_42_H_72_O_14_	801.024	HS	Saponin
C6	Ferulic Acid	C_10_H_10_O_4_	194.186	XCC	Organic acids
C7	Naringin	C_27_H_32_O_14_	580.539	XCC	Flavonoids
C8	Hesperidin	C_28_H_34_O_15_	610.565	XCC	Flavonoids
C9	Hesperetin	C_16_H_14_O_6_	302.282	XCC	Flavonoids
C10	Glaucocalyxin A	C_20_H_28_O_4_	332.44	XCC	Diterpenoids
C11	Oridonin	C_20_H_28_O_6_	364.438	XCC	Diterpenoid
C12	Quercetin	C_15_H_10_O_7_	302.238	XCC	Flavonoids
C13	Vitexin	C_21_H_20_O_10_	432.381	ZQ	Flavonoids

HS, Radix Ginseng Rubra; ZQ, *Fructus aurantii*; XCC, *Isodon amethystoides*.

**TABLE 5:** Potential protein targets of representative active compounds in WFC against PLGC.

ID	Full name of protein	Short name of protein
P1	Mitogen-activated protein kinase 1	MAPK1
P2	Arachidonate 5-lipoxygenase	ALOX5
P3	Macrophage migration inhibitory factor	MIF
P4	Catechol O-methyltransferase	COMT
P5	Prostaglandin G/H synthase 2	PTGS2
P6	Interleukin-1 beta	IL1B
P7	Interferon gamma	IFNG
P8	Interleukin-4	IL4
P9	Proheparin-binding EGF-like growth factor	HBEGF
P10	Caspase-3	CASP3
P11	RAC-alpha serine/threonine-protein kinase	AKT1
P12	Retinoblastoma-associated protein	RB1
P13	Mitogen-activated protein kinase 14	MAPK14
P14	Nuclear factor erythroid 2-related factor 2	NFE2L2
P15	Cytochrome P450 1A1	CYP1A1
P16	Protein kinase C beta type	PRKCB
P17	Pyruvate kinase PKM	PKM
P18	Vascular endothelial growth factor A	VEGFA
P19	Matrix metalloproteinase-9	MMP9
P20	Retinoblastoma-like protein 2	RBL2
P21	Cytochrome c	CYCS
P22	Cholecystokinin	CCK
P23	Dipeptidyl peptidase 4	DPP4
P24	Peroxisome proliferator-activated receptor gamma	PPARG
P25	Peroxisome proliferator-activated receptor alpha	PPARA
P26	NAD(P)H dehydrogenase [quinone] 1	NQO1
P27	Growth hormone secretagogue receptor type 1	GHSR
P28	B-cell lymphoma 6 protein	BCL6
P29	Cellular tumor antigen p53	TP53
P30	Glucose-6-phosphate 1-dehydrogenase	G6PD
P31	T-lymphocyte activation antigen CD80	CD80
P32	Heme oxygenase 1	HMOX1
P33	Sterol O-acyltransferase 1	SOAT1
P34	Cyclin-dependent kinase 2	CDK2
P35	Cyclin-dependent kinase 4	CDK4
P36	Neurogenic locus notch homolog protein 1	NOTCH1
P37	Tumor necrosis factor ligand superfamily member 6	FASLG
P38	Cytochrome P450 3A4	CYP3A4
P39	Nitric oxide synthase, inducible	NOS2
P40	Nitric oxide synthase, brain	NOS1
P41	Mitogen-activated protein kinase 8	MAPK8
P42	Poly [ADP-ribose] polymerase 1	PARP1
P43	NAD-dependent protein deacetylase sirtuin-1	SIRT1
P44	Cytochrome P450 1B1	CYP1B1
P45	Induced myeloid leukemia cell differentiation protein Mcl-1	MCL1
P46	Serine/threonine-protein kinase pim-1	PIM1
P47	Angiotensin-converting enzyme	ACE
P48	Hypoxia-inducible factor 1-alpha	HIF1A

#### Enrichment Analysis of Candidate Targets for Wei-Fu-Chun Against Precancerous Lesions of Gastric Cancer

**FIGURE 3:**
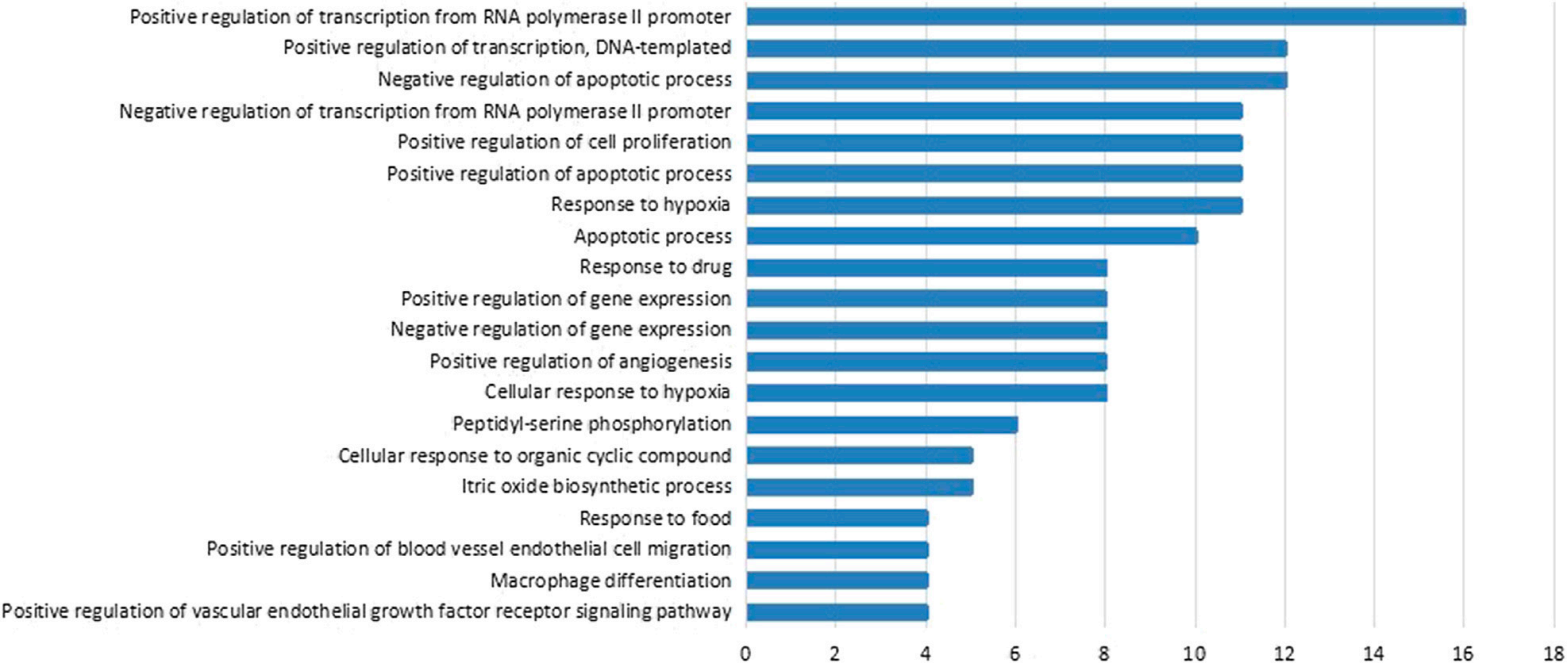
Biological processes regulated by WFC to treat PLGC. The count of each biological process was shown on the right side of the bar. The p-value of each biological process was less than 0.05. FDR of each biological process was less than 0.05.

To further explore the possible functions of the 48 therapeutic targets and reveal the relationship between active components and their underlying targets in PLGC, we performed pathway enrichment analysis on PLGC-related protein targets and obtained the main related signaling pathways of target proteins (*p*-value < 0.01). The 61 anti-PLGC pathways of WFC were listed in Table 6. These therapeutic targets were mainly distributed in PI3K/Akt pathway, MAPK pathway, vascular endothelial growth factor (VEGF) pathway, hypoxia-inducible factor-1 (HIF-1) pathway, and tumor necrosis factor (TNF) pathway, and FoxO pathway. In addition, According to the GO enrichment analysis results, we found that the functions of therapeutic targets were involved in multiple biological processes such as peptide chain serine phosphorylation, RNA polymerase II promoter transcription, cellular hypoxia, apoptosis, vascular endothelial cell migration, macrophage differentiation, vascular endothelial growth factor receptor, chemokine biosynthesis, cell proliferation, and nitric oxide biosynthesis (Figure 3).

**TABLE 6:** KEGG pathways regulated by WFC against PLGC.

NO.	Pathway ID	Pathway description	Count	Genes	*P*-Value
1	hsa05161	Hepatitis B	12	AKT1, MAPK1, CASP3, MMP9, CYCS, TP53, FASLG, MAPK8, RB1, CDK4, CDK2, PRKCB	1.77E−09
2	hsa05200	Pathways in cancer	17	PTGS2, MMP9, PPARG, CYCS, TP53, FASLG, RB1, CDK4, CDK2, PRKCB, AKT1, MAPK1, CASP3, HIF1A, VEGFA, MAPK8, NOS2	1.94E−09
3	hsa05219	Bladder cancer	7	MAPK1, MMP9, VEGFA, TP53, HBEGF, RB1, CDK4	2.41E−07
4	hsa05142	Chagas disease (American trypanosomiasis)	9	AKT1, MAPK1, ACE, MAPK14, IFNG, IL1B, FASLG, MAPK8, NOS2	3.38E−07
5	hsa05145	Toxoplasmosis	9	AKT1, MAPK1, CASP3, MAPK14, CYCS, IFNG, MAPK8, ALOX5, NOS2	5.21E−07
6	hsa05205	Proteoglycans in cancer	11	AKT1, MAPK1, CASP3, HIF1A, MAPK14, MMP9, VEGFA, TP53, HBEGF, FASLG, PRKCB	5.64E−07
7	hsa05222	Small cell lung cancer	8	AKT1, PTGS2, CYCS, TP53, RB1, NOS2, CDK4, CDK2	1.24E−06
8	hsa05206	MicroRNAs in cancer	12	NOTCH1, CASP3, CYP1B1, MCL1, PTGS2, HMOX1, MMP9, VEGFA, PIM1, TP53, SIRT1, PRKCB	1.92E−06
9	hsa04068	FoxO signaling pathway	9	AKT1, MAPK1, RBL2, MAPK14, FASLG, BCL6, MAPK8, SIRT1, CDK2	2.35E−06
10	hsa04066	HIF-1 signaling pathway	8	AKT1, MAPK1, HIF1A, HMOX1, VEGFA, IFNG, NOS2, PRKCB	2.84E−06
11	hsa05212	Pancreatic cancer	7	AKT1, MAPK1, VEGFA, TP53, MAPK8, RB1, CDK4	3.92E−06
12	hsa04668	TNF signaling pathway	8	AKT1, MAPK1, CASP3, PTGS2, MAPK14, MMP9, IL1B, MAPK8	5.88E−06
13	hsa05140	Leishmaniasis	7	IL4, MAPK1, PTGS2, MAPK14, IFNG, IL1B, NOS2	6.60E−06
14	hsa05164	Influenza A	9	AKT1, MAPK1, MAPK14, CYCS, IFNG, IL1B, FASLG, MAPK8, PRKCB	1.64E−05
15	hsa05152	Tuberculosis	9	AKT1, MAPK1, CASP3, MAPK14, CYCS, IFNG, IL1B, MAPK8, NOS2	1.86E−05
16	hsa05162	Measles	8	IL4, AKT1, IFNG, TP53, IL1B, FASLG, CDK4, CDK2	2.46E−05
17	hsa05223	Non-small cell lung cancer	6	AKT1, MAPK1, TP53, RB1, CDK4, PRKCB	3.17E−05
18	hsa04370	VEGF signaling pathway	6	AKT1, MAPK1, PTGS2, MAPK14, VEGFA, PRKCB	4.81E−05
19	hsa05210	Colorectal cancer	6	AKT1, MAPK1, CASP3, CYCS, TP53, MAPK8	5.21E−05
20	hsa05230	Central carbon metabolism in cancer	6	PKM, AKT1, MAPK1, HIF1A, G6PD, TP53	6.08E−05
21	hsa05214	Glioma	6	AKT1, MAPK1, TP53, RB1, CDK4, PRKCB	6.55E−05
22	hsa04664	Fc epsilon RI signaling pathway	6	IL4, AKT1, MAPK1, MAPK14, MAPK8, PRKCB	8.15E−05
23	hsa05133	Pertussis	6	MAPK1, CASP3, MAPK14, IL1B, MAPK8, NOS2	1.30E−04
24	hsa05168	Herpes simplex infection	8	CASP3, CYCS, IFNG, TP53, IL1B, FASLG, MAPK8, CDK2	1.87E−04
25	hsa05132	Salmonella infection	6	MAPK1, MAPK14, IFNG, IL1B, MAPK8, NOS2	2.11E−04
26	hsa04380	Osteoclast differentiation	7	AKT1, MAPK1, MAPK14, PPARG, IFNG, IL1B, MAPK8	2.13E−04
27	hsa04010	MAPK signaling pathway	9	AKT1, MAPK1, CASP3, MAPK14, TP53, IL1B, FASLG, MAPK8, PRKCB	2.32E−04
28	hsa05014	Amyotrophic lateral sclerosis	5	CASP3, NOS1, MAPK14, CYCS, TP53	3.22E−04
29	hsa05203	Viral carcinogenesis	8	PKM, MAPK1, CASP3, RBL2, TP53, RB1, CDK4, CDK2	3.75E−04
30	hsa04151	PI3K-akt signaling pathway	10	IL4, AKT1, MAPK1, MCL1, RBL2, VEGFA, TP53, FASLG, CDK4, CDK2	3.83E−04
31	hsa04932	Non-alcoholic fatty liver disease (NAFLD)	7	AKT1, PPARA, CASP3, CYCS, IL1B, FASLG, MAPK8	4.60E−04
32	hsa04660	T Cell receptor signaling pathway	6	IL4, AKT1, MAPK1, MAPK14, IFNG, CDK4	5.02E−04
33	hsa04620	Toll-like receptor signaling pathway	6	AKT1, MAPK1, CD80, MAPK14, IL1B, MAPK8	6.56E−04
34	hsa04210	Apoptosis	5	AKT1, CASP3, CYCS, TP53, FASLG	7.36E−04
35	hsa04919	Thyroid hormone signaling pathway	6	AKT1, MAPK1, NOTCH1, HIF1A, TP53, PRKCB	9.49E−04
36	hsa04115	p53 signaling pathway	5	CASP3, CYCS, TP53, CDK4, CDK2	9.86E−04
37	hsa04722	Neurotrophin signaling pathway	6	AKT1, MAPK1, MAPK14, TP53, FASLG, MAPK8	0.001150085
38	hsa04071	Sphingolipid signaling pathway	6	AKT1, MAPK1, MAPK14, TP53, MAPK8, PRKCB	0.001150085
39	hsa05218	Melanoma	5	AKT1, MAPK1, TP53, RB1, CDK4	0.001226145
40	hsa05169	Epstein-Barr virus infection	6	AKT1, MAPK14, TP53, MAPK8, RB1, CDK2	0.001238565
41	hsa05220	Chronic myeloid leukemia	5	AKT1, MAPK1, TP53, RB1, CDK4	0.001291966
42	hsa05332	Graft-vs.-host disease	4	CD80, IFNG, IL1B, FASLG	0.001326903
43	hsa05143	African trypanosomiasis	4	IFNG, IL1B, FASLG, PRKCB	0.001326903
44	hsa05160	Hepatitis C	6	AKT1, MAPK1, PPARA, MAPK14, TP53, MAPK8	0.001818362
45	hsa05330	Allograft rejection	4	IL4, CD80, IFNG, FASLG	0.001854692
46	hsa04914	Progesterone-mediated oocyte maturation	5	AKT1, MAPK1, MAPK14, MAPK8, CDK2	0.002598619
47	hsa04012	ErbB signaling pathway	5	AKT1, MAPK1, HBEGF, MAPK8, PRKCB	0.002598619
48	hsa04940	Type I diabetes mellitus	4	CD80, IFNG, IL1B, FASLG	0.002677073
49	hsa05215	Prostate cancer	5	AKT1, MAPK1, TP53, RB1, CDK2	0.002709164
50	hsa04912	GnRH signaling pathway	5	MAPK1, MAPK14, HBEGF, MAPK8, PRKCB	0.003060147
51	hsa04913	Ovarian steroidogenesis	4	CYP1B1, CYP1A1, PTGS2, ALOX5	0.004158486
52	hsa04723	Retrograde endocannabinoid signaling	5	MAPK1, PTGS2, MAPK14, MAPK8, PRKCB	0.004453312
53	hsa05231	Choline metabolism in cancer	5	AKT1, MAPK1, HIF1A, MAPK8, PRKCB	0.004453312
54	hsa05146	Amoebiasis	5	CASP3, IFNG, IL1B, NOS2, PRKCB	0.005288502
55	hsa04621	NOD-like receptor signaling pathway	4	MAPK1, MAPK14, IL1B, MAPK8	0.006056193
56	hsa04726	Serotonergic synapse	5	MAPK1, CASP3, PTGS2, ALOX5, PRKCB	0.006223084
57	hsa00140	Steroid hormone biosynthesis	4	CYP3A4, CYP1B1, CYP1A1, COMT	0.006678959
58	hsa04650	Natural killer cell mediated cytotoxicity	5	MAPK1, CASP3, IFNG, FASLG, PRKCB	0.008653181
59	hsa04110	Cell cycle	5	RBL2, TP53, RB1, CDK4, CDK2	0.009153158
60	hsa05211	Renal cell carcinoma	4	AKT1, MAPK1, HIF1A, VEGFA	0.009543869
61	hsa05120	Epithelial cell signaling in Helicobacter pylori infection	4	CASP3, MAPK14, HBEGF, MAPK8	0.009945007

#### Construction of Pharmacology-Network for Wei-Fu-Chun Against Precancerous Lesions of Gastric Cancer

**FIGURE 4:**
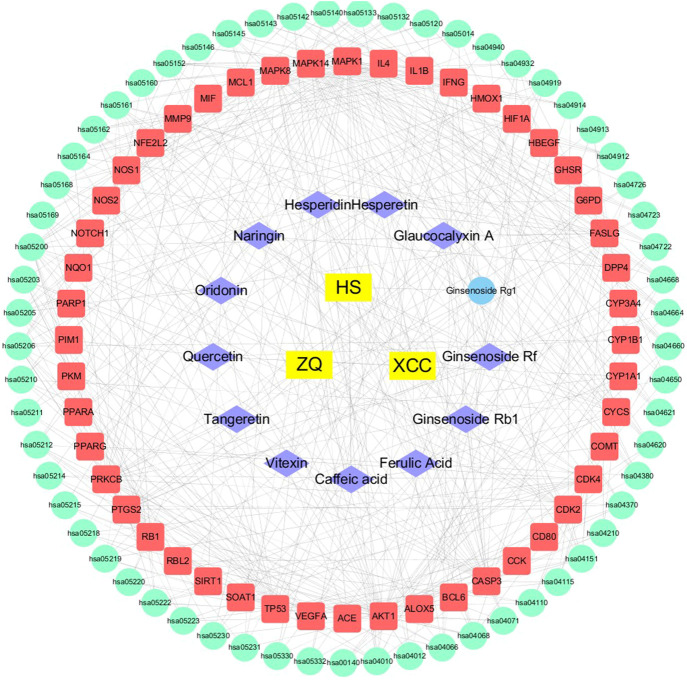
Pharmacology-network of the “origins-components-targets-pathways” regulated by WFC. The yellow rectangles represent Chinese herbal medicines, while the purple rhombuses represent active components of WFC. The blue ellipse represents metabolites of ginsenoside Rg1. The red rectangles represent target proteins, and the green ellipses represent pathways in **Table 6**.

Using the Cytoscape 3.1 software, we constructed an “origins-components-targets-pathways” pharmacology network of WFC (Figure 4). This network depicted the relationship between 61 pathways, 48 therapeutic targets, 13 active components and corresponding plant materials. The network also consisted of 64 nodes and 75 edges, of which Ginsenoside Rb_1_ (C3, degree = 7), Naringin (C7, degree = 9), Hesperidin (C8, degree = 8), Hesperetin (C9, degree = 7), and Oridonin (C11, degree = 8) had high degrees and were centrally located in the network, suggesting that these active compounds may be the key components of WFC in the treatment of PLGC.

### Model Test Results of Precancerous Lesions of Gastric Cancer

**FIGURE 5:**
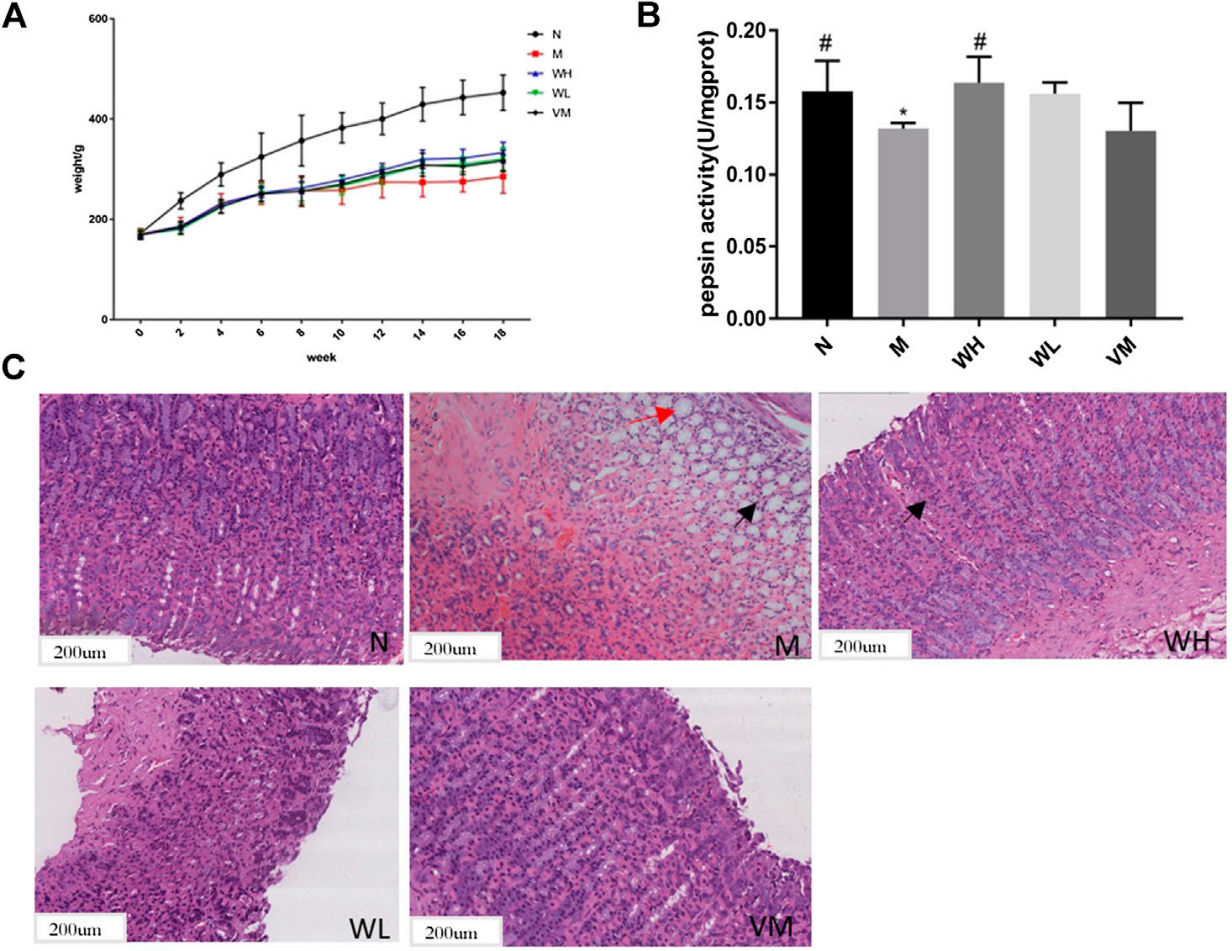
Pharmacodynamic effect of WFC on PLGC models. **(A)** The effect of WFC on the weight change of rats with PLGC. **(B)** Pepsin activity changes in gastric tissue of rats in different groups. **(C)** Pathological changes of gastric tissue of in different groups of rats (H&E: 100×). *p < 0.05 compared with the normal group; ^#^*p* < 0.05 compared with the model group.

**FIGURE 6:**
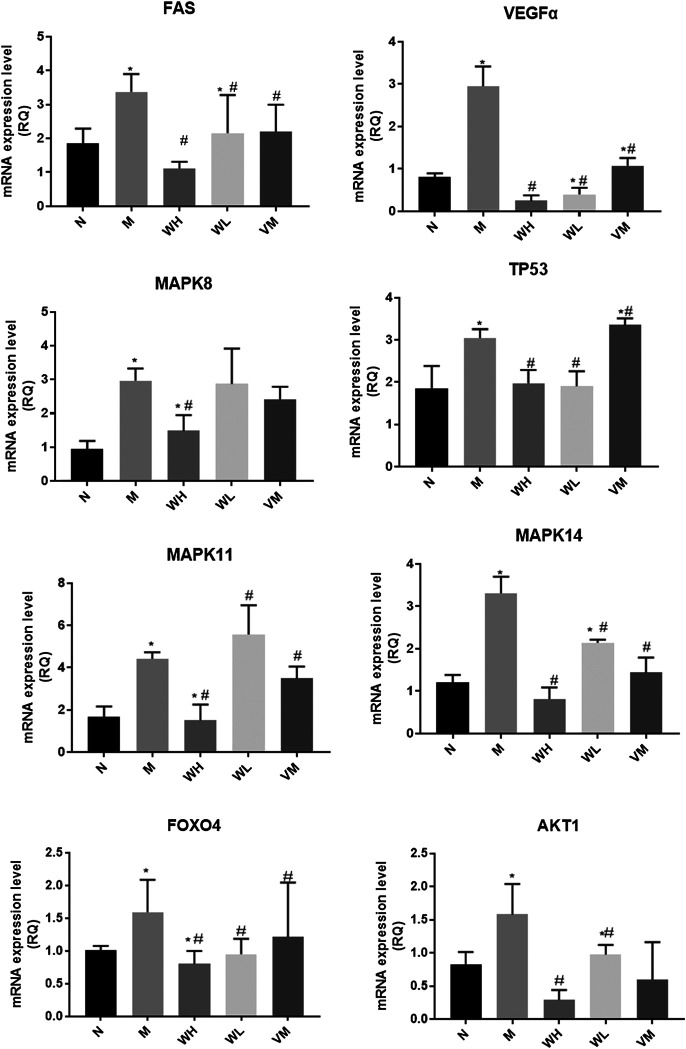
Gene expression related to MAPK pathways in gastric tissues of different groups of rats. *p < 0.05 compared with the normal group; ^#^*p* < 0.05 compared with the model group.

The speed of weight gain in the model group was much lower than that in the normal group. At the end of the fourth week, the weight of the high dose group, the low dose group, and the Vitacoenzyme group showed a rising trend after the simultaneous modeling and administration of the high dose group, the low dose group, and the vitamin enzyme group (Figure 5A). The increase rate of the high dose group was higher than that of the other two groups. The weight of the model group increased and then decreased until the end of the model. Compared with the normal group, there was a significant statistical difference in the activity value of pepsin in the normal group and the model group (*p* < 0.05). Compared with the model group, the activity value of pepsin in the high-dose group was significantly different (*p* < 0.05). There was no significant difference in protease activity between the low dose group and the model group. There was also no significant difference in pepsin activity between the high-dose group and the normal group (*p* > 0.05) (Figure 5B). The histopathology of gastric mucosa was demonstrated by hematoxylin eosin staining. Atrophic glands, intestinal metaplasia and lymphocyte infiltration were shown in the model group, and compared with the model group, the above pathological manifestations were alleviated in varying degrees (Figure 5C). RT-PCR results showed that mRNA expression of VEGF, FOXO4, AKT, TP53, FAS, MAPK8, MAPK11, and MAPK14 in the model group was significantly up-regulated compared with that in the N group (*p* < 0.05)., while mRNA expression of interleukin-10 (interleukin-10, il-10) was down-regulated (*p* < 0.01). mRNA expressions of VEGF, FOXO4, AKT, TP53, FAS, MAPK8, MAPK11, and MAPK14 were all significantly down-regulated in the WFC high dose group (*p* < 0.05) (Figure 6).

## Discussion

WFC, an herbal prescription with three medicines, may contain thousands of compounds, however, naringin is the only maker compound of WFC in 2015 edition of “Chinese Pharmacopoeia.” In this study, we hypothesized that unilateral factors and single targets are insufficient to demonstrate the complex mechanisms of WFC. The network pharmacology method used in this study is a novel methodology based on the construction of multilayer networks of disease phenotype-gene-drug to predict drug targets in a holistic manner and promote efficient drug discovery (Liu and Sun, 2012). This method represents a breakthrough in comparison with the traditional herbal medicine research pattern “gene target-disease” and initiates the new pattern of “multiple genes multiple targets-complex diseases” (Hopkins, 2008; Ma et al., 2016). With this method, we proved that XCC was the critical ingredient involved in the treatment of PLGC, which corresponded to the constituents of WFC with 86.8% weight of XCC in WFC. HS was also a major herb that regulated PLGC. Moreover, WFC is a multiple-component complex system. (Zhang et al., 2012) unambiguously identified or tentatively characterized 46 components in WFC tablet, including 26 saponins, 10 flavonoids, and 10 other compounds. However, it is not enough to understand the chemical components of WFC, which makes it rather difficult to define the functions of this herbal medicine from material basis and chemical properties. Therefore, a chemical fingerprint analysis of WFC was necessary. Compared with the material basis of the three herbs in WFC, a chemical fingerprint analysis of WFC was fully carried out by means of UHPLC-ESI-Q-TOF/MS. Finally, a total of 178 compounds were identified in WFC, including 93 compounds (70 terpene) originally from *I. amethystoides*, 51 compounds from *Fructus aurantii*, 31 compounds from red ginseng, which basically demonstrated the material basis in WFC. However, the main components’ content and dose-effect relationship should be further investigated.

In network pharmacology studies, there were 86 candidate compounds (77 absorbed components and nine metabolites) and 105 potential targets. Thirteen active components and 48 protein targets were selected to explore the effect of WFC against PLGC. The results showed that PI3K/Akt pathway, MAPK pathway, VEGF pathway, HIF-1 pathway, TNF pathway, and FOXO pathway may be involved as the anti-PLGC pathways of WFC. In addition, ginsenoside Rb_1_, naringin, hesperidin, hesperetin, and oridonin may be the key components of WFC in the treatment of PLGC. Ginsenoside Rb1was shown to exert anti-inflammatory effects in Modulating TLR-4 dimerization and NF-kB/MAPKs signaling pathways (Gao et al., 2020). Ginsenoside Rb_1_ also enhanced the phagocytic capacity of macrophages for bacteria via activation of the p38/Akt pathway, which may be a useful pharmacological adjuvant for the treatment of bacterial infections in clinically relevant conditions (Xin et al., 2019). Moreover, naringin induced autophagy-mediated growth inhibition by downregulating the PI3K/Akt/mTOR cascade via activation of MAPK pathways in AGS cancer cells (Raha et al., 2015). As for hesperetin, it induced apoptosis in human glioblastoma cells via p38 MAPK activation (Li et al., 2020). Finally, oridonin’s anticancer effects on colon cancer were mediated via BMP7/p38 MAPK/p53 signaling (Liu et al., 2018). Overall, this suggested that MAPK pathway, PI3K/Akt pathway, and p38/Akt pathway may be the key pathways of WFC treating PLGC.

To verify the network pharmacology prediction results, *in vivo* rat experiments were carried out. Pepsin is formed by pepsinogen stimulated by gastric acid. Pepsin is mainly secreted by the main cells. Atrophy of gastric glands and metaplasia of intestinal epithelium reduce the main cells. From superficial gastritis, atrophic gastritis/dysplasia to gastric cancer, pepsinogen in pathological tissues decreases and then pepsin activity decreases. In chronic atrophic gastritis patients with or without intestinal metaplasia, pepsinogen I often decreased (Terasawa et al., 2014). In this experiment, WFC restored pepsin activity in the rat model of PLGC.

MAPK is the main transmitter of intracellular and extracellular signals. P38 and JNK are the important parts of its four major subgroups. JNK pathway and p38 pathway mediate the transmission of cytokines and inflammatory mediators and participate in cell cycle, apoptosis, and migration. This process plays an important role in the development of precancerous lesions to gastric cancer. JNK mainly exists in the cytoplasm and accumulates rapidly and significantly in the nucleus after being activated by the superior kinase. The activation of transcription factors in the nucleus includes TP53, c-Jun and so on (Liu and Lin, 2005), and then produces biological effects. The positive rate of JNK expression in gastric cancer was 75%, and it positively correlated with the size of the tumor and the early and late stage of the tumor (Wang, 2009). *Helicobacter pylori* can stimulate the invasion of AGS gastric cancer cells through JNK signaling pathway (Díaz-Serrano et al., 2018). Inhibition of JNK pathway can enhance the antitumor effect of trail on MGC803 gastric cancer cells (Liu et al., 2011). P38 has five isomers, p38 α (p38), p38 ß 1, p38 ß 2, p38 γ, p38 δ. 38 α, and p38 ß tissues are widespread. P38 pathway plays an important role in cell cycle regulation and can induce cell cycle. The inhibition of p38 pathway may be one of the mechanisms of synergistic antitumor effect of Adriamycin and monomer PA-2 (Liang et al., 2020). In gastric cancer patients with high expression of lncRNA-aoc4p, inhibiting the expression of lncRNA-aoc4p could reduce the expression level of JNK and p38 protein and inhibit cell proliferation, migration, and invasion (Qu et al., 2019). FOXO4, as a member of the FOXO family of transcription factors, is regulated by microRNA in a variety of cancer cells, and its abnormal expression is closely related to gastrointestinal tumors (Gross et al., 2008; Liu et al., 2020). The current study further proved the therapeutic effect of WFC on PLGC in rats, and preliminarily explored and verified the results of network pharmacology. The detection results of MAPK pathway genes p38 α, p38 β, JNK and its upstream and downstream factors TP53 and VEGF α, as well as FOXO4 and AKT genes showed consistency. We believe that MAPK pathway is involved in the mechanism of action of WFC on PLGC, which needs to be further explored.

## Conclusion

In conclusion, a simple and reliable UHPLC–ESI-Q-TOF/MS technique was established to profile complex compounds in WFC and describe their absorption behavior in plasma samples after oral administration of WFC. A total of 178 compounds were identified. In addition, 77 absorbed compounds of parent molecules in WFC were detected. Among these compounds, the most absorbed ones were terpenes from XCC, flavonoids from ZQ and saponins from HS. The current study supplemented previous WFC research, and MS data contributed to the identification of allied natural compounds. The screening of *in vivo* absorbed and metabolic compounds provided constructive material basis for further research on the pharmacology and curative mechanisms of WFC. Moreover, the network pharmacology method was used to predict the active components, corresponding therapeutic targets, and related pathways of WFC in the treatment of PLGC. Finally, based on the major compounds of WFC and their metabolites in rat plasma and existing databases, 13 active components, 48 therapeutic targets, and 61 pathways were found to act against PLGC. The results from rat experiment showed that WFC could improve the weight of PLGC rats and the gastric mucosa histopathological changes partly by inhibiting MAPK signaling pathway to increase pepsin secretion.

This study offered an applicable approach for the identification of chemical components, absorbed compounds, and metabolic compounds of WFC, and provided a method to explore bioactive ingredients and action mechanisms of WFC.

## Data Availability Statement

The raw data supporting the conclusions of this manuscript will be made available by the authors, without undue reservation, to any qualified researcher.

## Author Contributions

HW, RW, and DX contributed equally. HW conducted the experimental part of the main component analysis of WFC. Network pharmacology was mainly analyzed by RW, DX performed most of the pharmacological experiments, analyzed the data, and participated in the manuscript draft. LD and XL helped complete animal feeding and analysis of the main components in plasma of Weifuchun tablets. YB, XC, and BN polished the manuscript. SW, KL, and WC monitored the drug quality and helped complete the data collation. GY and MS directed the study, and drafted and finalized the manuscript. All authors read and approved the final manuscript.

## Funding

This work was supported by Science and Technology Commission of Shanghai Municipality (Nos. 15DZ1900104 and 15DZ1900100 to MS and GY); the fourth batch of Chinese medicine (basic) talents of the State Administration of TCM (No. 2017-124 to MS); Construction of Postgraduate Innovation Course in Shanghai University of Traditional Chinese Medicine (No. 2017 to MS); and Natural Science Foundation of Shanghai (No. 20ZR1458100 to HW).

## Conflict of Interest

HW, RW, XL, KL and GY were employed by Shangai Pharmaceuticals Holding Co., Ltd.. LD was employed by Shanghai Zhonghua Pharmaceuticals Co., Ltd. WC were employed by Huqingyutang Chinese Medicine Medernization Research Institute of Zhejiang Province. The remaining authors declare that the research was conducted in the absence of any commercial or financial relationships that could be constructed as a potential conflict of interest.
